# Asymmetric Division and Differential Gene Expression during a Bacterial Developmental Program Requires DivIVA

**DOI:** 10.1371/journal.pgen.1004526

**Published:** 2014-08-07

**Authors:** Prahathees Eswaramoorthy, Peter W. Winter, Peter Wawrzusin, Andrew G. York, Hari Shroff, Kumaran S. Ramamurthi

**Affiliations:** 1 Laboratory of Molecular Biology, National Cancer Institute, National Institutes of Health, Bethesda, Maryland, United States of America; 2 Section on High Resolution Optical Imaging, National Institute of Biomedical Imaging and Bioengineering, National Institutes of Health, Bethesda, Maryland, United States of America; Max Planck Institute for Terrestrial Microbiology, Germany

## Abstract

Sporulation in the bacterium *Bacillus subtilis* is a developmental program in which a progenitor cell differentiates into two different cell types, the smaller of which eventually becomes a dormant cell called a spore. The process begins with an asymmetric cell division event, followed by the activation of a transcription factor, σ^F^, specifically in the smaller cell. Here, we show that the structural protein DivIVA localizes to the polar septum during sporulation and is required for asymmetric division and the compartment-specific activation of σ^F^. Both events are known to require a protein called SpoIIE, which also localizes to the polar septum. We show that DivIVA copurifies with SpoIIE and that DivIVA may anchor SpoIIE briefly to the assembling polar septum before SpoIIE is subsequently released into the forespore membrane and recaptured at the polar septum. Finally, using super-resolution microscopy, we demonstrate that DivIVA and SpoIIE ultimately display a biased localization on the side of the polar septum that faces the smaller compartment in which σ^F^ is activated.

## Author Summary

A central feature of developmental programs is the establishment of asymmetry and the production of genetically identical daughter cells that display different cell fates. Sporulation in the bacterium *Bacillus subtilis* is a simple developmental program in which the cell divides asymmetrically to produce two daughter cells, after which the transcription factor σ^F^ is activated specifically in the smaller cell. Here we investigated DivIVA, which localizes to highly negatively curved membranes, and discovered that it localizes at the asymmetric division site. In the absence of DivIVA, cells failed to asymmetrically divide and prematurely activated σ^F^ in the predivisional cell, largely unreported phenotypes for any deletion mutant in a sporulation gene. We found that DivIVA copurifies with SpoIIE, a protein that is required for asymmetric division and σ^F^ activation, and that both proteins preferentially localize on the side of the septum facing the smaller daughter cell. DivIVA is therefore a previously overlooked structural factor that is required at the onset of sporulation to mediate both asymmetric division and compartment-specific transcription.

## Introduction

**Figure 1:**
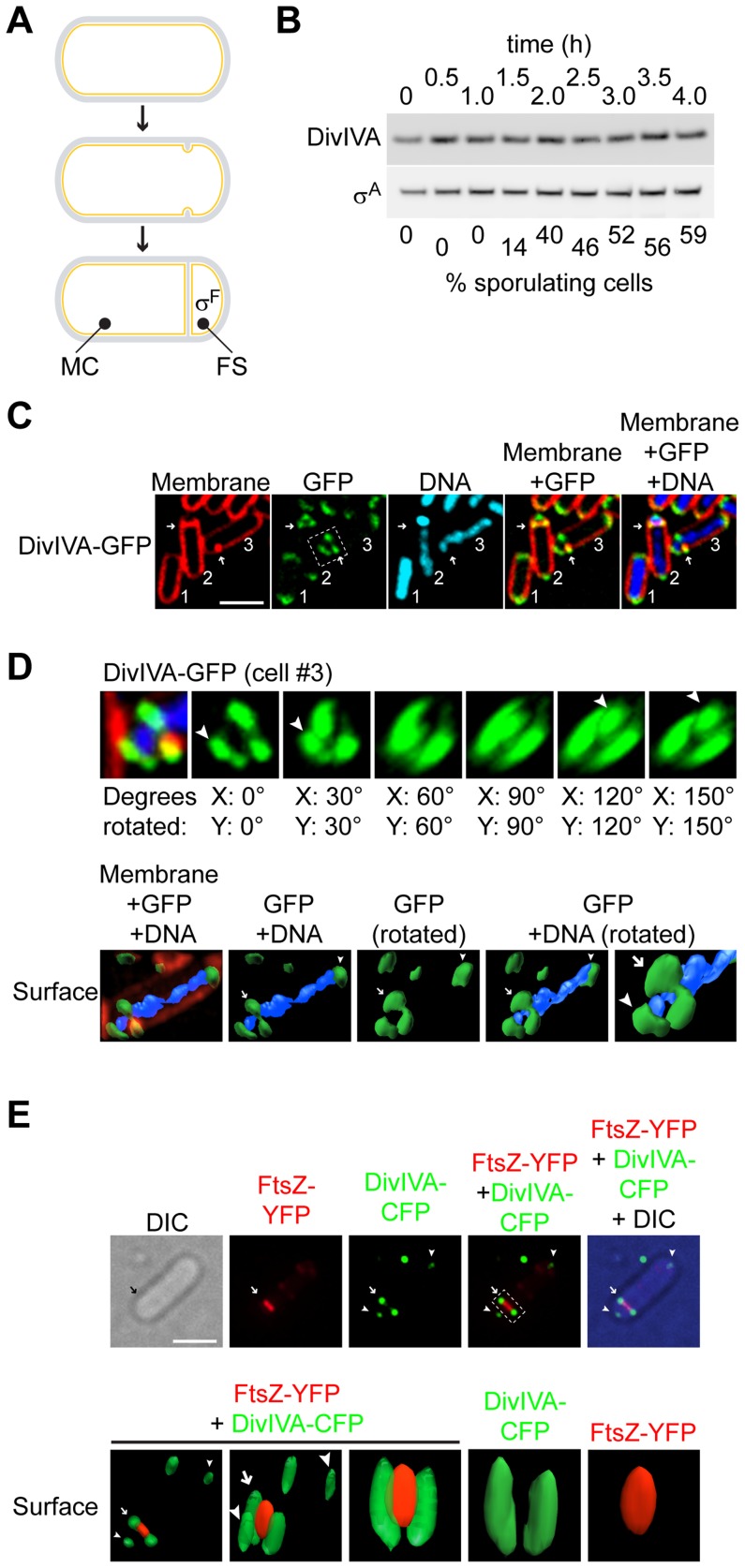
DivIVA assembles into a ring-like structure at the polar septum during sporulation. (A) Schematic representation of sporulation in *Bacillus subtilis*. Depicted is a progenitor rod-shaped cell (above), the onset of asymmetric septation (middle), and formation of the polar septum (below), where the mother cell (MC) and forespore (FS) are labeled; activation of σ^F^ exclusively in the forespore is also depicted. (B) Immunoblot analysis of wild type cells (strain PY79; for genotypes, see Table S1), induced to sporulate and harvested at the times indicated above, using antisera specific to DivIVA or σ^A^ (an abundant unrelated protein that served as a loading control). Fraction of cells that had entered sporulation, as determined by epifluorescence microscopy, is indicated below. (C) Localization of DivIVA-GFP in strain KR604 induced to sporulate for 1.5 h. First panel: membranes were visualized using the fluorescent dye FM4-64; second panel: fluorescence from GFP; third panel: chromosomes were visualized using DAPI; fourth panel: overlay of membrane and GFP; fifth panel: overlay of membrane, GFP, and DNA. Cell #1 is a pre-divisional cell; cell #2 has elaborated a polar septum; cell #3 has just initiated asymmetric division, as evidenced by an increase in membrane staining at the future site of septation. Arrows indicate the sites of asymmetric division. (D) Top: rotation of the region of cell #3 indicated in (C), second panel, around the x- and y-axes. Degrees rotated are indicated below each panel; arrowheads indicate DivIVA patches. Bottom: GFP and DAPI fluorescence from cell #3 (C) represented as a three-dimensional surface. Arrow indicates DivIVA ring at the polar septum; arrowheads indicate the same DivIVA patches at poles. (E) Top: Localization of DivIVA-CFP (shown in green) and FtsZ-YFP (shown in red) in strain PE177. First panel: differential interference contrast (DIC); second panel: fluorescence from YFP; third panel: fluorescence from CFP; fourth panel: overlay of YFP and CFP; fifth panel: overlay of YFP, CFP, and DIC. Bottom: YFP and CFP fluorescence from the cell shown above represented as a three-dimensional surface and rotated to view at different angles. Panels 1–3: overlay of YFP and CFP. Arrow indicates DivIVA-CFP ring or constricting FtsZ-YFP at the polar division site; arrowheads indicate DivIVA-CFP patches at the extreme poles of the cell. Scale bars: 2 µm.

Asymmetric cell division and differential gene expression are hallmarks that underlie the differentiation of a progenitor cell into two genetically identical, but morphologically dissimilar daughter cells [1]–[5]. The rod shaped Gram-positive bacterium *Bacillus subtilis*, which normally divides by binary fission to produce two identical daughter cells, undergoes such a differentiation program, termed sporulation, when it senses the imminent onset of starvation conditions (reviewed in [6]–[8]). During sporulation, *B. subtilis* first divides asymmetrically by elaborating a so-called “polar septum” that produces two unequal-sized daughter cells: a larger “mother cell” and a smaller “forespore” (Fig. 1A) that each receive one copy of the genetic material. After asymmetric division, the daughter cells remain attached and a compartment-specific transcription factor called σ^F^ is exclusively activated in the forespore. This activation step is critical because it sets off a cascade of transcription factor activation events, each in an alternating compartment, resulting in the expression of a unique set of genes in each daughter cell, which ultimately drives the rest of the sporulation program [9], [10]. Subsequently, the forespore is engulfed by the mother cell and eventually the forespore achieves a partially dehydrated state of dormancy in which its metabolic activity is largely arrested and is released into the environment when the mother cell ultimately lyses- the released cell is termed a “spore” (or, formally, an “endospore”) [11]. Several factors that are required for the switch from medial to asymmetric division have been identified, but the mechanisms underlying this switch remain largely unknown. Similarly, the biochemical basis for the activation of σ^F^ has been well elucidated, but the cell biological basis for how this activation is achieved exclusively in the forespore is less well known.

At the onset of sporulation, FtsZ, the bacterial tubulin homolog that provides the force for membrane invagination during cytokinesis, initially assembles at mid-cell into a ring-like structure called the “Z-ring” [12]–[14]. At this time, an integral membrane protein called SpoIIE is also produced in the pre-divisional cell and co-localizes with FtsZ via a direct interaction [15]–[17]. Instead of constricting at mid-cell, though, the Z-ring next unravels and extends outward towards each pole via a helix-like intermediate and finally reassembles as two separate Z-rings near the two poles of the bacterium; SpoIIE similarly redeploys to the two polar positions with FtsZ [18]. This redeployment of the Z-ring requires SpoIIE and increased expression of *ftsZ* from a second sporulation-specific promoter [18]–[23]. Next, one of the two polar Z-rings constricts [24], [25], thereby elaborating the polar septum on one end of the bacterium. Although FtsZ constricts at this site and eventually dissipates into the cytosol, SpoIIE somehow remains associated with the polar septum [15], [16], [26]–[28]. A recent report demonstrated that SpoIIE is released into the forespore membrane soon after septum formation is complete and that it is recaptured at the polar septum [29]. Interestingly, the total level of SpoIIE before release and after recapture was similar suggesting that SpoIIE is exclusively released into the forespore membrane after septum formation, but the mechanism by which FtsZ could preferentially release SpoIIE into the forespore membrane is not known.

After formation of the polar septum SpoIIE performs a second function in which it activates σ^F^ [23], [30]. Prior to asymmetric division, σ^F^ is synthesized in the pre-divisional cell, but is held inactive by an anti-sigma factor called SpoIIAB [31], [32]. After asymmetric septation, SpoIIE, whose C-terminus harbors a phosphatase domain [33], [34], dephosphorlyates an anti-anti-sigma factor (SpoIIAA), which then binds and sequesters SpoIIAB, thereby relieving σ^F^ inhibition [23], [35]–[37]- somehow, this activity is manifested only in the forespore compartment. Some evidence has suggested that this compartment exclusivity is ultimately due to a preferential localization of SpoIIE on the forespore side of the polar septum [38], [39], but how and when this asymmetric localization initially arises has not been clear.

In this study, we examined the subcellular localization of DivIVA, a peripheral membrane protein made of coiled-coil domains that spontaneously assembles into a higher order structure, at the onset of asymmetric division. During vegetative growth of *B. subtilis*, DivIVA localizes to nascent cell division sites at mid-cell at the very onset of membrane constriction [40]. It has been proposed that negative membrane curvature, such as that which arises on either side of a division septum where it meets the lateral edge of the cell, provides a geometric cue that drives the localization of DivIVA to assemble into ring-like structures on both sides of a division septum [40]–[43]. During normal growth, DivIVA rings serve as platforms that recruit the MinCD complex [40], [44]–[46], which inhibits FtsZ assembly, to either side of the nascent cell division septum [47]. As a result, aberrant FtsZ assembly immediately adjacent to a newly forming septum (and thus, the formation of “minicells” devoid of DNA) is inhibited and membrane constriction occurs once, and only once, at mid-cell [40], [47]. At the onset of sporulation, DivIVA performs a second function: DivIVA rings collapse into patches at the two hemispherical cell poles [40] where it anchors the origins of replication of the two replicated chromosomes (via a DNA-binding protein called RacA) [41], [48]–[50], thereby assuring that both the forespore and mother cell receive one copy of the chromosome.

Here, we report that DivIVA localizes to the polar septum and plays an additional role at the onset of sporulation. Deletion of the *divIVA* gene or depletion of DivIVA protein after its chromosome anchoring function resulted in a severe asymmetric septation defect due to an inability of cells to redeploy FtsZ and SpoIIE from medial to polar positions. As a result, cells arrested at this stage of sporulation, unlike other division mutations reported to cause an asymmetric division defect, prematurely activated σ^F^ in a compartment-unspecific manner. We discovered that DivIVA and SpoIIE exist in a complex with one another in sporulating cells and that, when co-produced in vegetative cells, SpoIIE did not persist at division septa in the absence of DivIVA, consistent with a model in which DivIVA is required to briefly anchor SpoIIE at the polar septum during sporulation once FtsZ begins to constrict and subsequently leaves the septum, before SpoIIE is released into the forespore membrane. Finally, employing super-resolution microscopy, we observed that DivIVA initially localized to both sides of the polar septum at the very onset of membrane invagination, but that it preferentially persisted at the forespore side once septation was completed. In contrast, SpoIIE preferentially localized to the forespore side of the polar septum at the onset of membrane constriction, and maintained this biased localization pattern until the completion of polar septum formation. Eventually, SpoIIE was released into the forespore membrane. We propose that DivIVA performs a previously unappreciated role in asymmetric division and compartment-specific activation of σ^F^ during sporulation.

## Results

### DivIVA assembles into a ring-like structure at the asymmetric septum

DivIVA was previously reported to localize to medially-placed division septa that form during vegetative growth in *Bacillus subtilis*, but not to the asymmetrically-placed “polar” septum that forms at the onset of sporulation [50]. This implied that DivIVA is somehow able to detect a feature that is exclusive to vegetative division septa, and is absent at asymmetric septa. DivIVA, however, reportedly localizes to division septa by detecting a geometric localization cue, not a chemical cue such as a pre-localized protein: namely, it is thought to preferentially embed onto negatively curved (concave) patches of membrane, such as those that are present where a division septum meets the lateral edge of a rod-shaped cell [41], [42]. Since negative membrane curvature is a feature that is shared by medially-placed division septa that are formed during vegetative growth and asymmetrically-placed polar septa during sporulation, we hypothesized that, if present during sporulation, DivIVA should indeed also localize to the polar septum in sporulating cells. To verify if DivIVA is present at the time of asymmetric septation, we first observed the total levels of DivIVA protein at various time points in synchronized cultures of sporulating *B. subtilis* by immunoblotting with antibodies specific to DivIVA. Entry into the sporulation pathway was followed by epifluorescence microscopy and enumerating the number of cells that had elaborated a polar septum. Although not all cells in the culture typically initiate sporulation, by 2.5 hours after initiation of the program approximately half of the cells had elaborated a polar septum, yet DivIVA protein levels were largely unchanged, and remained constant for at least the first four hours after induction of sporulation, suggesting that DivIVA persists well into sporulation (Fig. 1B). To examine the subcellular localization of DivIVA in sporulating cells, we first constructed a DivIVA-GFP fusion with a flexible linker that had been previously used to construct a nearly fully functional DivIVA-CFP fusion [44], produced under the control of its native promoter at the ectopic, non-essential *amyE* locus. As reported for the CFP fusion, cells harboring DivIVA-GFP as the only copy of DivIVA were of similar size as wild type cells and produced very few minicells, suggesting that the fusion protein is largely functional (Fig. S1). The localization of DivIVA-GFP to the polar septum was observed in 93% of sporulating cells that we examined (n = 105; Fig. 1C; the remaining 7% of cells counted displayed either no or weak DivIVA-GFP signal). Reconstruction of deconvolved Z-stacks of a cell that was beginning to elaborate a polar septum (cell #3, Fig. 1C) revealed that DivIVA formed a ring-like structure (Fig. 1D, top row), similar to DivIVA ultrastructure found at vegetative septa as reported previously [40], [43]. Representation of the reconstructed fluorescence signal of DivIVA-GFP and DAPI (indicating the chromosome) as a surface revealed two separate populations of DivIVA: one at the extreme poles that fulfills the chromosome anchoring role of DivIVA patches, and a second, ring-like localization of DivIVA at the nascent polar septum through which the chromosome was threaded (Fig. 1D, bottom row). Time lapse epifluorescence microscopy revealed that soon after formation of the polar septum initiated, DivIVA-GFP localized at that site and remained associated with the completed septum (Fig. S2B).

Next, we examined if the DivIVA ring remains at the site of septation even after membrane constriction (similar to what has been observed at vegetative septa) or if it collapses with the rest of the division machinery as cytokinesis proceeds. We observed the localization of DivIVA-CFP and FtsZ (the major component of the divisome that drives membrane constriction [51]), fused to YFP, in sporulating cells that produced both proteins and had just initiated asymmetric septation. In these cells, FtsZ-YFP localized as a band whose width was less than the width of the entire cell (Fig. 1E, top row), consistent with a pattern of constriction of the division machinery. In the same cells, though, DivIVA-CFP remained as two foci at the site of division that did not overlap with the fluorescence signal from FtsZ-YFP, consistent with the formation of a static ring that did not constrict. Reconstruction of deconvolved Z-stacks revealed that FtsZ-YFP formed a collapsing disk-like structure during constriction of the nascent polar septum, whereas DivIVA-CFP remained as a ring-like structure with an outer diameter that was larger than that of FtsZ-YFP (1E, bottom row). We conclude that, similar to the situation at vegetative septa, DivIVA localizes to the polar septum at the onset of sporulation and remains, at least initially, at that site even after elaboration of the septum.

### DivIVA is required for asymmetric division

To study the role that DivIVA may play at the polar septum, we sought to monitor sporulation in cells harboring a deletion of the *divIVA* gene using fluorescence microscopy. However, Δ*divIVA* cells are severely elongated relative to wild type cells during vegetative growth and display a severe sporulation defect, presumably because the morphology of the cells is so different. Moreover, DivIVA is required at the onset of sporulation to anchor the origins of the replicated chromosomes to the two poles via an anchoring protein called RacA, thereby rendering the straightforward cytological analysis of a simple Δ*divIVA* mutant during sporulation difficult. We therefore engineered a strain in which DivIVA could be proteolytically degraded after its role during vegetative growth and chromosome anchoring had finished, but before the polar septum was elaborated. To this end, *divIVA* at its native locus was replaced with *divIVA-FLAG* fused to an altered *ssrA* peptide tag named *ssrA^Ec^*. Additionally, the *sspB* gene from *E. coli*, which encodes an adaptor that specifically delivers SsrA^Ec^-tagged proteins to the ClpXP proteolytic machinery, was produced under the control of an inducible promoter from an ectopic site on the chromosome, so that DivIVA-FLAG-SsrA^Ec^ could be specifically degraded upon addition of inducer [52]. The dimensions of cells harboring *divIVA-FLAG-ssrA^Ec^* were similar to that of wild type (Fig. S1), indicating that this allele of *divIVA* was functional. Finally, to identify individual cells that had properly entered the sporulation pathway, GFP was produced in this strain under the control of a sporulation-specific promoter (*P_spoIIG_*).

**Figure 2:**
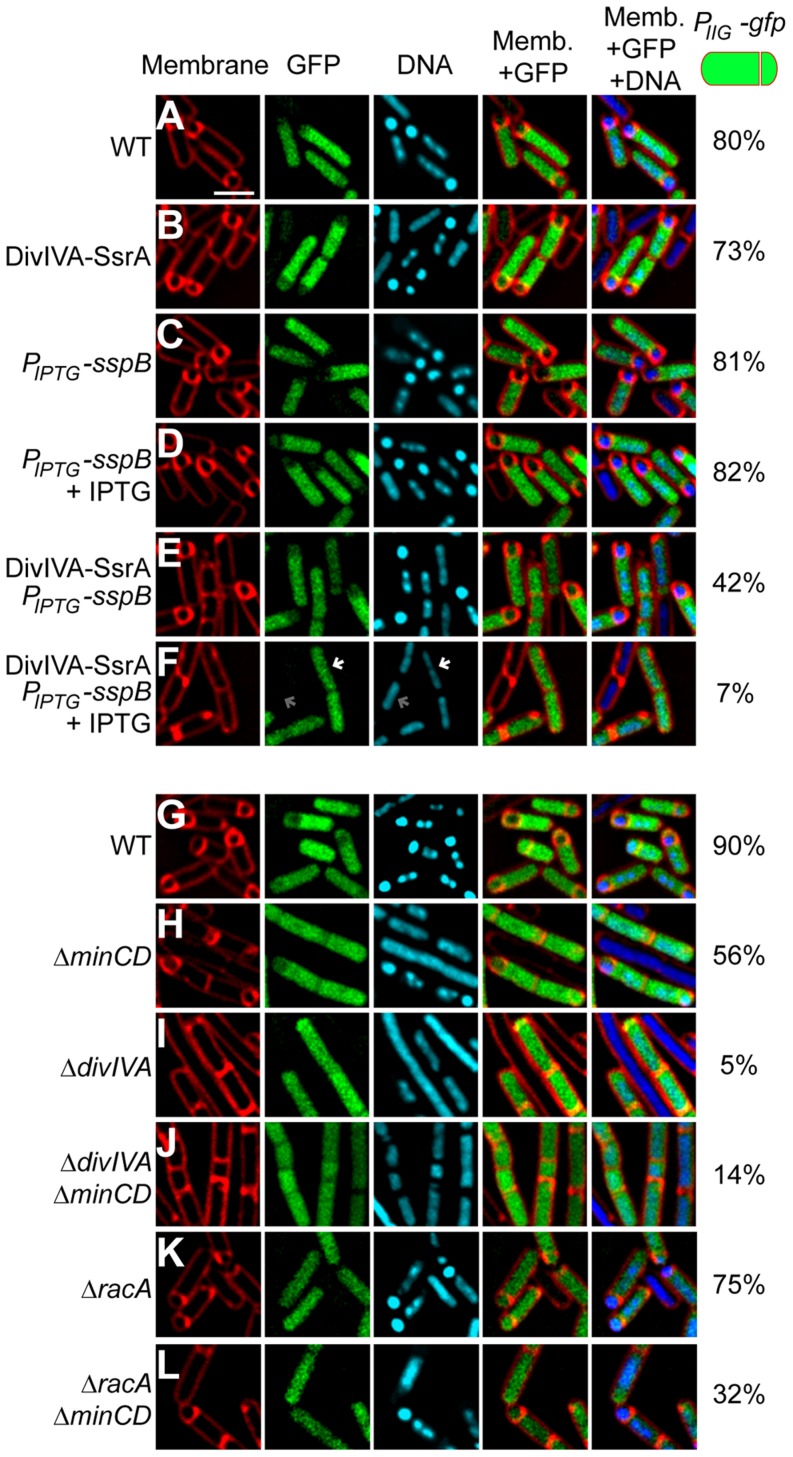
DivIVA is required for asymmetric division during sporulation. (A–F) Polar septum formation was monitored using the fluorescent membrane dye FM4-64 in cells that had initiated sporulation (evidenced by production of GFP under control of the sporulation-specific promoter *P_spoIIG_*) for 2 h in (A) otherwise wild type cells (strain PE303); (B) cells expressing *divIVA-ssrA^Ec^* (strain PE304); expressing *sspB* alone under an IPTG-inducible promoter (strain PE329) in the absence (C) or presence (D) of IPTG; or co-expressing *divIVA-ssrA^Ec^* and *sspB* (strain PE330) in the absence (E) or presence (F) of IPTG. First panel: membranes visualized using FM4-64; second panel: GFP fluorescence indicating sporulating cells; third panel: chromosomes visualized using DAPI; fourth panel: overlay of membrane and GFP; fifth panel: overlay of membrane, GFP, and DNA. Fraction of cells that had initiated sporulation (evidenced by production of GFP) and had elaborated a polar septum is indicated to the right. White arrow in (F) indicates condensed chromosome in a GFP-producing cell; gray arrow indicates an uncondensed chromosome in a non-sporulating cell. (G–L) Polar septum formation was monitored in sporulating cells producing GFP as described above in otherwise (G) wild type cells (strain PE305); (H) Δ*minCD* (strain PE306); (I) Δ*divIVA* (strain PE307); (J) Δ*divIVA* Δ*minCD* (strain PE318); (K) Δ*racA* (strain PE339); or (L) Δ*racA*Δ*minCD* (strain PE340). Scale bar: 2 µm.

Two hours after the induction of sporulation, 80% of otherwise wild type cells (n = 140) had both produced GFP (indicating that they had initiated sporulation) and had elaborated a polar septum (Fig. 2A); similar fractions of cells harboring *divIVA-ssrA^Ec^* as the only allele of *divIVA* (73%, n = 142; Fig. 2B) or *sspB* only, in the absence or presence of the inducer IPTG, (81%, n = 124; and 82%, n = 189, respectively; Fig. 2C–D) both entered the sporulation pathway and elaborated polar septa, indicating that neither the SsrA^Ec^ tag on DivIVA, nor the presence of the SspB adaptor affected entry into sporulation or asymmetric division. When expression of *sspB* was induced 45 minutes after the synchronized induction of sporulation, DivIVA-SsrA^Ec^ was largely undetectable in 15 minutes (Fig. S3; compare t60 between +IPTG and −IPTG). Surprisingly, when examined by fluorescence microscopy, only 7% of the cells that had initiated sporulation (evidenced by production of GFP; n = 147) elaborated a polar septum. Interestingly, these cells also displayed a condensed and elongated chromosome architecture as evidenced by DAPI staining that appeared untethered at the poles, similar to that observed in a Δ*racA* strain ([48]; Fig. 2F: compare the cell marked with white arrow and gray arrow), suggesting that the classically defined “Stage I” of sporulation (in which chromosomes replicate once and condense) had been achieved, but that “Stage II”, in which the polar septum is formed, was blocked. In the absence of inducer, only 42% of these cells (n = 172; Fig. 2E) elaborated a polar septum (we suspect due to leaky expression of *sspB* that led to overall reduced levels of DivIVA-SsrA^Ec^ as seen in Fig. S3), suggesting that SspB-mediated degradation of DivIVA-FLAG-SsrA^Ec^ blocked asymmetric division.

To eliminate the possibility that this observed defect in polar septation was an unrelated consequence of the strategy involving the timed degradation of DivIVA-FLAG-SsrA^Ec^, we repeated the experiment in strains harboring a deletion of *divIVA*, but whose elongation phenotype was suppressed by the additional deletion of *minCD* [48], [50], [53]. Again, to monitor cells that had entered the sporulation pathway, we introduced a GFP reporter produced under the control of a sporulation promoter. Whereas 90% (n = 120) of otherwise wild type cells and 56% (n = 114) of Δ*minCD* cells producing GFP formed asymmetric septa (Fig. 2G–H), only 5% (n = 126) of Δ*divIVA* cells and 14% (n = 140) of Δ*divIVA* Δ*minCD* cells producing GFP elaborated asymmetric septa (Fig. 2I–J), indicating that the absence of DivIVA resulted in an asymmetric division defect. To test if this defect was due to the inability of RacA to interact with DivIVA, we examined asymmetric septation in cells harboring a deletion in *racA*. The absence of RacA alone had a modest asymmetric septation defect (Fig. 2K; 75% septation, n = 100). Deletion of both *racA* and *minCD* reduced the frequency of asymmetric septation to 32% (n = 125; Fig. 2L), possibly due to an additive effect of removing both MinCD and RacA function, but still the defect was not as severe as the Δ*divIVA* defect. Taken together, we conclude that DivIVA plays a previously unappreciated role in the asymmetric placement of the polar septum at the onset of sporulation and that in the absence of DivIVA, cells are arrested at the classically defined “Stage I” of sporulation in which chromosome condensation occurs, but polar septation is prevented.

### DivIVA is required for the deployment of FtsZ from medial to polar position

**Figure 3:**
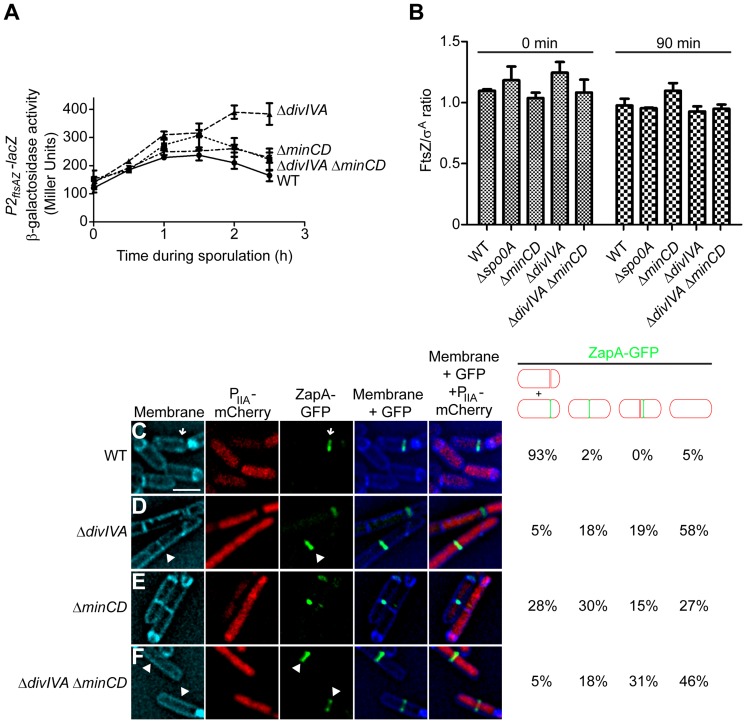
DivIVA is required for the deployment of FtsZ to polar sites. (A) β-galactosidase accumulation was measured at different time points after the induction of sporulation in cells harboring a *P2_ftsAZ_-lacZ* reporter fusion in otherwise wild type cells (•; strain MF3595), Δ*minCD* (▪; strain PE294), Δ*divIVA* (▴; strain PE295), or Δ*divIVA* Δ*minCD* (▾; strain PE296). (B) Steady state levels of FtsZ, relative to the constitutively produced protein σ^A^, as determined by immunoblot analysis using antisera specific to FtsZ or σ^A^ of cell extracts prepared after the induction of sporulation at the times indicated from the following strains: wild type (strain PY79), Δ*spo0A* (strain PE362), Δ*minCD* (strain KR620), Δ*divIVA* (strain KR543), and Δ*minCD* Δ*divIVA* (strain PE308). Error bars represent standard deviation from the mean from three independent trials; immunoblots used for the analysis are shown in Fig. S4A. (C–F) Localization of ZapA-GFP in cells also producing mCherry under control of a sporulation-specific promoter (*P_spoIIA_*) harvested two hours after the induction of sporulation in (C) an otherwise wild type strain (strain PE292); (D) Δ*divIVA* (strain PE320); (E) Δ*minCD* (strain PE319); or (F) Δ*divIVA* Δ*minCD* (strain PE325). Membranes were visualized with TMA-DPH. Arrows indicate ZapA-GFP at polar division sites; arrowheads indicate ZapA-GFP at midcell. Fraction of sporulating cells (i.e., producing mCherry) that had elaborated a polar septum, displaying ZapA-GFP at the proper asymmetric division site, mid-cell, near mid-cell, or not displaying ZapA-GFP at all, are indicated to the right. Scale bar: 2 µm.

At the onset of sporulation, FtsZ, the bacterial tubulin homolog that drives membrane constriction during cytokinesis, initially assembles as a ring at mid-cell, but then redeploys towards the two poles and forms two polar-localized rings, at which time only one ring constricts to form the polar septum. This redeployment requires an increase in expression of the *ftsZ* gene [18], which is mediated by the activation of a second sporulation-specific promoter (“*P*_2_”) that is dependent on the Spo0A transcription factor, the master regulator of entry into sporulation [21]. To test if DivIVA affects expression levels of *ftsZ*, we monitored *ftsZ* transcription by placing the *lacZ* gene, which encodes β-galactosidase, under the control of the *P*_2_ promoter of *ftsZ* and measured at different time points the β-galactosidase activity in synchronized sporulating cells that harbored this construct. β-galactosidase activity of the *P_2_* promoter reached its peak between 1 h–1.5 h after the induction of sporulation in otherwise wild type cells (Fig. 3A). In cells harboring a deletion of either *minCD* alone or *divIVA* and *minCD*, the profile of β-galactosidase was nearly identical to that of wild type cells, and β-galactosidase activity in Δ*divIVA* cells even continued to rise after t_1.5_, suggesting that the asymmetric septation defect in Δ*divIVA* Δ*minCD* cells is not due to the absence of the transcription burst of *ftsZ* during sporulation. Next, we checked the steady state levels of FtsZ protein by immunoblotting cell extracts prepared from various strains of *B. subtilis*. At the time that sporulation was induced steady state FtsZ protein levels relative to σ^A^ in the absence of MinCD, DivIVA, or MinCD and DivIVA were similar to that of wild type, and remained similar even 90 min after the induction of sporulation (Fig. 3B, Fig. S4A). Although the burst in transcriptional activity did not evidently result in a sustained increase in steady state levels of FtsZ protein (measured at a population level at the time points that we tested), the data nonetheless indicate that the steady state levels of FtsZ protein were not reduced in the absence of DivIVA, and that the failure of FtsZ rings to redeploy in the absence of DivIVA is not due to a reduction in FtsZ level.

To test if the asymmetric septation defect in the absence of DivIVA was due to the failure of FtsZ to redeploy to polar sites or due to the inability of FtsZ rings to constrict after redeployment, we monitored the subcellular localization of the division machinery in sporulating cells. To avoid complications arising from the co-production of natively produced FtsZ and ectopically produced FtsZ-GFP, we examined the localization of an FtsZ-associated protein that promotes FtsZ assembly (ZapA) fused to GFP that is frequently used as a proxy for FtsZ localization [54], [55]. ZapA-GFP robustly re-deploys to the polar septum approximately 60 min after the induction of sporulation (Fig. S4B). To identify cells that had initiated sporulation, we also introduced a cassette that expressed *mCherry* under the control of an early sporulation-specific promoter (*P*_spoIIA_). Additionally, to ensure that Δ*divIVA* mutants may not simply be slightly delayed in elaborating polar septa, we observed all cells two hours after the induction of sporulation (at a time when polar septation was usually completed in wild type cells and engulfment was initiating) and enumerated the total number of cells harboring either a completed septum or a polar-localized ZapA-GFP. In otherwise wild type cells, two hours after the induction of sporulation, polar septa were elaborated, or were about to be elaborated, as evidenced by ZapA-GFP localization at a polar position (Fig. 3C, arrow; Fig. S4B), in 93% of cells (n = 100) that had produced mCherry. In Δ*divIVA* cells, only 5% of sporulating cells produced a polar septum (Fig. 3D), but in Δ*minCD* cells, approximately one third of cells either elaborated a polar septum or displayed ZapA-GFP at a polar site (Fig. 3E). In Δ*divIVA* Δ*minCD* cells, though, only 5% of cells that initiated sporulation elaborated polar septa (Fig. 3F) at this time point. In almost half of these sporulating cells, ZapA-GFP remained at mid-cell (18%) or immediately adjacent to mid-cell (31%) and had not redeployed to a polar site (Fig. 3F). In the remaining 46%, no ZapA-GFP structure was detected, suggesting that the protein was diffusely localized in the cytosol. We conclude that the asymmetric septation defect in the absence of DivIVA is due to the failure of FtsZ to redeploy and assemble into Z-rings at polar sites.

### DivIVA interacts with SpoIIE at the polar septum

**Figure 4:**
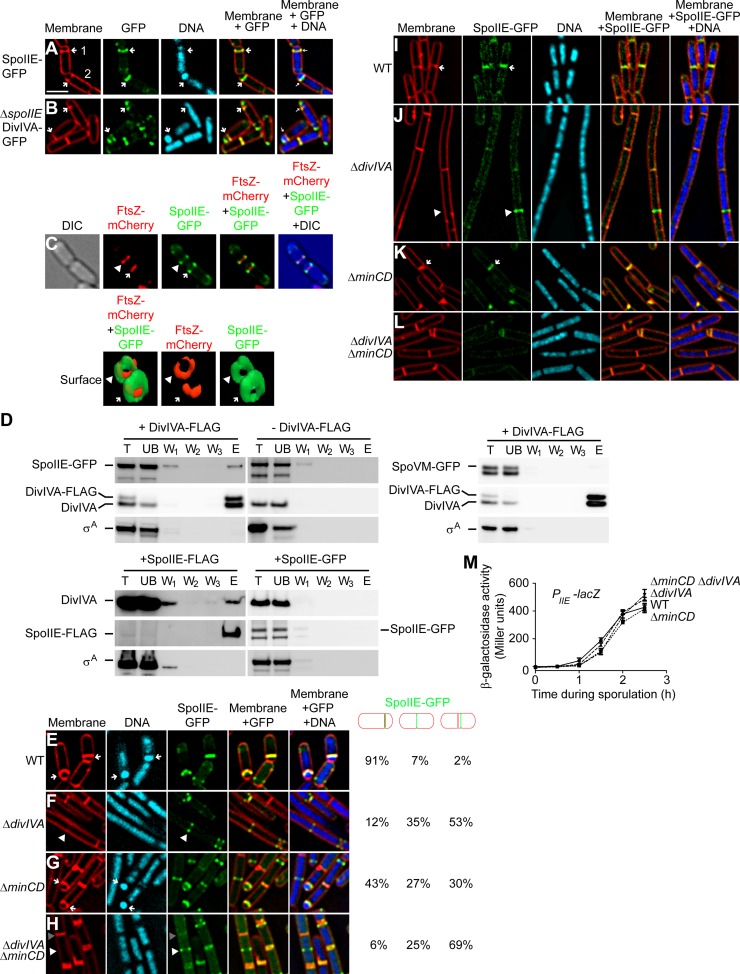
SpoIIE copurifies with DivIVA and proper SpoIIE localization requires DivIVA. (A) Localization of SpoIIE-GFP in sporulating cells (strain PE118). (B) Localization of DivIVA-GFP in sporulating cells in the absence of SpoIIE (strain PE266). First panel: membranes visualized using FM4-64; second panel: GFP fluorescence; third panel: chromosomes visualized using DAPI; fourth panel: overlay of membrane and GFP; fifth panel: overlay of membrane, GFP, and DNA. Polar division sites are indicated with arrows. (C) Top: localization of FtsZ-mCherry and SpoIIE-GFP at polar septa of sporulating cells (strain PE369). First panel: DIC; second panel: mCherry fluorescence; third panel: GFP fluorescence; fourth panel: overlay of mCherry and GFP; fifth panel: overlay of DIC, mCherry, and GFP. Bottom: GFP and mCherry fluorescence represented as a three-dimensional surface. Arrowhead indicates nascent polar septum before FtsZ constriction; arrow indicates a polar division site at which FtsZ is constricting. (D) Co-immunoprecipitation experiment of DivIVA with SpoIIE. Total detergent-solublized extracts (T) prepared from sporulating cells producing DivIVA-FLAG and SpoIIE-GFP (top left, strain PE148), only SpoIIE-GFP (top right, strain PE118), DivIVA-FLAG and SpoVM-GFP (middle, strain PE388), SpoIIE-FLAG (bottom left, strain PE375), or SpoIIE-GFP (bottom right, strain PE130) were incubated with resin covalently attached to anti-FLAG antibodies. Unbound (UB) material was removed, the resin was washed extensively (W_1_–W_3_), and bound material was eluted (E) using excess FLAG peptides. Fractions were analyzed by immunoblotting using antisera specific to GFP, DivIVA, or σ^A^. (E–H) Localization of SpoIIE-GFP produced under control of its native promoter in cells harvested 90 min after the induction of sporulation: (E) Otherwise wild type (strain PE118), (F) Δ*divIVA* (strain PE122), (G) Δ*minCD* (strain PE138), or (H) Δ*divIVA* Δ*minCD* (strain PE141). Fraction of cells in which SpoIIE-GFP was at a polar division site, at mid-cell, or adjacent to mid-cell are indicated to the right. First panel: membranes visualized using FM4-64; second panel: chromosomes visualized using DAPI; third panel: GFP fluorescence; fourth panel: overlay of membrane and GFP; fifth panel: overlay of membrane, GFP, and DNA. Arrows indicate forespores; white arrowheads indicate SpoIIE-GFP at a nascent cell division site before elaboration of a septum; gray arrowheads indicate the absence of SpoIIE-GFP at a mature septum. (I–L) Localization of SpoIIE-GFP produced under control of an inducible promoter in vegetatively growing cells: (I) otherwise wild type (strain PE130), (J) Δ*divIVA* (strain PE133), (K) Δ*minCD* (strain PE224), or (L) Δ*divIVA* Δ*minCD* (strain PE225). First panel: membranes visualized using FM4-64; second panel: GFP fluorescence; third panel: chromosomes visualized using DAPI; fourth panel: overlay of membrane and GFP; fifth panel: overlay of membrane, GFP, and DNA. Arrows indicate SpoIIE at mature septa; white arrowheads indicate SpoIIE-GFP at a nascent cell division site before elaboration of a septum; gray arrowheads indicate the absence of SpoIIE-GFP at a mature septum. (M) β-galactosidase accumulation was measured at different time points after the induction of sporulation in cells harboring a P*_spoIIE_-lacZ* reporter fusion in otherwise wild type cells (•; strain PE301), Δ*minCD* (▪; strain PE323), Δ*divIVA* (▴; strain PE324), or Δ*divIVA* Δ*minCD* (▾; strain PE328). Scale bars: 2 µm.

Since DivIVA typically forms a platform to recruit other proteins to particular subcellular sites (MinJ to division septa, or RacA to the cell poles [44], [45], [48], [56]), we speculated that DivIVA could anchor a sporulation protein at the polar septum. Given the asymmetric septation defect caused by the absence of DivIVA, we wondered if DivIVA could influence the activity of SpoIIE, a transmembrane protein that is involved first in shifting the division septum from the medial to the polar site at the onset of sporulation; and then in activating the first compartment-specific sigma factor, σ^F^, specifically in the forespore [23], [30]. A functional SpoIIE-GFP fusion initially localizes as a ring at mid-cell, likely via a direct interaction with FtsZ, and then redeploys to polar sites with FtsZ ([18]; Fig. 4A; Fig. S1D). However, unlike FtsZ, which constricts during septation and eventually dissipates in the cytosol after membrane constriction is finished, SpoIIE remains at the polar septum until septum formation is complete and is then redistributed throughout the forespore membrane to perform its second function in σ^F^ activation [29]. However, the mechanism by which it initially persists at this site is not known [15], [26], [27], [29]. In 66% of observed sporulating cells (n = 161), SpoIIE-GFP persisted roughly uniformly along the entire length of the polar septum. In the remaining 34%, we observed two separate populations of SpoIIE at the polar septum: one near the center of the polar septum and another that remained near the lateral edge of the cell (Fig. 4A, cell 1). To see if SpoIIE co-constricts with FtsZ at the polar septum, we examined the localization of both proteins in sporulating cells producing FtsZ-mCherry and SpoIIE-GFP. At the onset of asymmetric division, both FtsZ-mCherry and SpoIIE-GFP co-localized as bands whose widths were approximately equal to the width of the cell (Fig. 4C, top row, arrowhead). However, as FtsZ constricted to form the polar septum, FtsZ-mCherry localized as a band whose width was less than the width of the cell, whereas in 91% of these observed cells (n = 100) SpoIIE-GFP remained as a band whose width was approximately equal to the width of the cell (Fig. 4C, arrow). Reconstruction of deconvolved Z-stacks revealed that, before FtsZ constriction initiated, both FtsZ-mCherry and SpoIIE-GFP assembled into ring-like structures (Fig. 4C, bottom row, arrowhead), but upon constriction of FtsZ at the polar septum, FtsZ-mCherry formed a disk-like structure, whereas SpoIIE-GFP remained as a ring-like structure with an outer diameter that was larger than that of FtsZ-mCherry (Fig. 4C, arrow). This localization pattern of SpoIIE was similar to that observed for DivIVA-CFP at the polar septum (Fig. 1E) and consistent with a model in which FtsZ constricts, while DivIVA and SpoIIE remain associated (SpoIIE albeit briefly) at the lateral edge of the polar septum.

To test if SpoIIE and DivIVA interact in vivo, we constructed a cell that produced, under the control of their native promoters, a functional DivIVA with a C-terminally appended FLAG tag (Fig. S1) in addition to the untagged version of DivIVA; as well as SpoIIE-GFP (also functional in vivo; (Fig. S1D; [27]). We then purified DivIVA-FLAG, using anti-FLAG antibodies, from detergent-solubilized cell extracts and examined various fractions collected during purification by immunoblotting. Although DivIVA localizes to the membrane, its association with the membrane is likely tenuous [57]; accordingly it appeared largely in the soluble fraction of cell extracts even without the inclusion of detergent (Fig. S5). However, the polytopic membrane protein SpoIIE was initially insoluble, but was effectively solubilized by addition of detergent (Fig. S5). The results in Fig. 4D indicate that SpoIIE-GFP co-purified with DivIVA-FLAG, as did the native, untagged version of DivIVA. As a negative control, the unrelated protein σ^A^ was not retained on the column. To ensure that an unrelated membrane-associated protein did not co-purify with DivIVA-FLAG, we repeated the experiment using a strain that overproduced the membrane-associated protein SpoVM-GFP, and observed that SpoVM-GFP also did not co-purify with DivIVA-FLAG. Furthermore, when the purification was performed with cell extract which did not produce DivIVA-FLAG, neither SpoIIE-GFP nor DivIVA was retained on the column, suggesting that retention of SpoIIE-GFP was specifically dependent on the presence of the FLAG-tagged DivIVA. Finally, we performed the reciprocal pulldown experiment in which we purified functional SpoIIE-FLAG (Fig. S1D) and observed that DivIVA, but not σ^A^, co-purified with SpoIIE-FLAG. As a negative control, when the SpoIIE-GFP was purified with anti-FLAG antibodies, SpoIIE-GFP, DivIVA, and σ^A^ were not retained on the column, suggesting that the specific co-purification of DivIVA was mediated by SpoIIE-FLAG. We therefore conclude that SpoIIE and DivIVA interact with each other in vivo and that this interaction likely takes place at the polar septum.

To test if DivIVA localization at the polar septum is dependent on SpoIIE, we examined the localization of DivIVA-GFP in sporulating cells in the absence of SpoIIE. Even though SpoIIE is involved in shifting the septum formation site to the polar position during sporulation, deletion of *spoIIE* does not completely abolish asymmetric division and about 30% of the cells elaborate a polar septum [18]. In cells harboring a deletion of *spoIIE*, DivIVA-GFP localization was unaffected and it continued to localize at the polar septum (Fig. 4B). Taken together, we conclude that DivIVA is in complex with SpoIIE, and that the localization of DivIVA to the polar septum does not depend on SpoIIE.

Next, we examined the subcellular localization of SpoIIE-GFP, produced under control of its native promoter, in the presence and absence of DivIVA. In otherwise wild type cells, SpoIIE was found at the polar septum or at a potential site of asymmetric septation in 91% of cells 1.5 h after the induction of sporulation (Fig. 4E). Δ*divIVA* cells, though, due to the defect in polar septation, displayed SpoIIE-GFP in only 12% of cells (Fig. 4F). Once polar septation was restored by deletion of *minCD*, nearly half of the cells displayed SpoIIE-GFP at the polar septum (Fig. 4G). However, in Δ*divIVA* Δ*minCD* cells, again due to the defect in polar septation, SpoIIE-GFP was found at the polar septum in only 6% of cells (Fig. 4H). Rather, like the localization of ZapA (and by extension, FtsZ), in a majority of cells SpoIIE-GFP was observed either at mid-cell (25%) or at a site immediately adjacent to mid-cell (69%). To ensure that the SpoIIE localization defect in the absence of DivIVA was not due to a defect in transcription levels of the *spoIIE* gene, we placed the *lacZ* gene under the control of the *spoIIE* promoter and measured β-galactosidase activity in sporulating cells at different time points. The results in Fig. 4M indicated *spoIIE* transcription was largely unaffected in cells harboring a deletion in *divIVA*, *minCD*, or both, as compared to wild type. Thus, in the absence of DivIVA, SpoIIE failed to redeploy from mid-cell to its customary polar positions.

### Persistence of SpoIIE at vegetative septa is dependent on DivIVA

Does the interaction between SpoIIE and DivIVA play a role in transiently sequestering SpoIIE at the polar septum? Whereas the dependence of DivIVA-GFP localization on SpoIIE was readily measured by deleting the *spoIIE* gene (Fig. 4B), the converse experiment was not straightforward to perform, since deletion of *divIVA* resulted in the failure to elaborate polar septa (Fig. 4H). We therefore produced SpoIIE-GFP under the control of an inducible promoter in vegetative cells, in the absence of other sporulation factors, and examined its localization either in the presence or absence of DivIVA. When produced in vegetatively growing wild type cells, SpoIIE localized to division septa and persisted at 82% (n = 146) of mature septa after cytokinesis had completed ([26]; Fig. 4I). It should be noted, however, that we observed the eventual release of SpoIIE from mature septa (unlike DivIVA) in wild type cells after approximately 2–3 cell generations, suggesting that an interaction between SpoIIE and DivIVA, even in vegetative cells, may be transient. In cells harboring a *divIVA* deletion, SpoIIE readily localized to future division sites (presumably dependent on FtsZ), but persisted at only 12% (n = 122) of mature septa (Fig. 4J). To ensure that this reduction in localization was not due to the infrequent septum formation, we suppressed the cell elongation phenotype of the Δ*divIVA* strain by introducing a deletion in *minCD* [48], [50], [53]. In Δ*minCD* Δ*divIVA* cells, cells were approximately of wild type length and septa were elaborated much more frequently. In these cells, SpoIIE-GFP still failed to persist at mature septa (19%, n = 100; Fig. 4L). As a control, the deletion of *minCD* alone did not abolish the persistence of SpoIIE at division septa (43%, n = 100; Fig. 4K). Taken together, we conclude that although DivIVA is not required for the initial recruitment of SpoIIE to the future site of cell division (consistent with a model in which FtsZ initially recruits SpoIIE [16], [17]), the transient persistence of SpoIIE at mature septa after cytokinesis has finished depends on DivIVA.

### DivIVA is required for the compartment-specific activation of σ^F^

**Figure 5:**
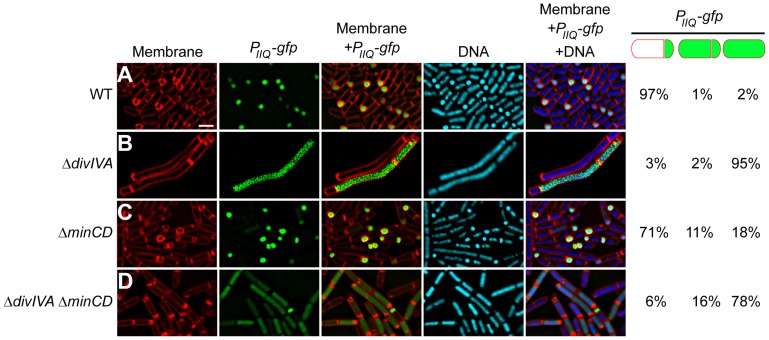
σ^F^ is promiscuously mis-activated in the absence of DivIVA. Compartment specificity of σ^F^ activation was monitored using fluorescence microscopy in cells producing GFP under control of the forespore-specific *P_spoIIQ_* promoter in (A) otherwise wild type cells (strain PE80), (B) Δ*divIVA* (strain PE81), (C) Δ*minCD* (strain PE259), or (D) Δ*divIVA* Δ*minCD* (strain PE260). First panel: membranes visualized using FM4-64; second panel: GFP fluorescence; third panel: overlay of membrane and GFP; fourth panel: chromosomes visualized using DAPI; fifth panel: overlay of membrane, GFP, and DNA. Fraction of asymmetrically divided cells either displaying forespore-specific or promiscuous production of GFP, and pre-divisional cells displaying promiscuous production of GFP are indicated to the right. Scale bar: 2 µm.

To verify if the absence of DivIVA affects the second function of SpoIIE, which is to activate σ^F^ specifically in the forespore, we used a strain in which *gfp* was under the control of a σ^F^-controlled promoter (*P_spoIIQ_*) and monitored the forespore-specific production of GFP in the presence or absence of DivIVA. In an otherwise wild type strain, 97% (n = 131) of cells that produced GFP produced it exclusively in the forespore (Fig. 5A). In cells harboring a deletion of *divIVA*, of the cells that produced GFP, 95% (n = 108) of them displayed uncompartmentalized activation of σ^F^ (Fig. 5B). Since Δ*divIVA* cells are morphologically so dissimilar to sporulating wild type cells, we also examined σ^F^ activation in Δ*minCD* Δ*divIVA* cells. In this strain, only 6% of GFP-producing cells displayed forespore-specific activation of σ^F^ (n = 106), whereas deletion of *minCD* alone resulted in proper forespore-specific σ^F^ activation in 71% (n = 143) of the cells (Fig. 5C–D), suggesting that DivIVA is required for compartment-specific activation of σ^F^. This unspecific activation of σ^F^ in the absence of DivIVA was unlike the phenotype seen in the absence of other division factors, such as FtsZ, FtsA, DivIC, and FtsL, in which asymmetric division was impaired, but σ^F^ activation was prevented as well [58]–[61]. Analysis of a σ^F^ responsive promoter (*P_spoIIQ_*) fused to *lacZ* indicated that the total amount of σ^F^ activity at a population level was not significantly affected in the absence of DivIVA (Fig. S6A). Thus, the absence of DivIVA instead primarily affected the compartment specificity of σ^F^ activation.

A previous study had reported that a *spoIIE^V697A^* mutant allele caused premature activation of σ^F^, which in turn resulted in an asymmetric septation defect in that strain [62]. Does the uncompartmentalized activation of σ^F^ in the absence of DivIVA, then, result in the asymmetric septation defect that we observed in these strains? If so, then deletion of *sigF* should correct the asymmetric septation defect in the absence of DivIVA. We therefore introduced a *sigF* deletion in strains that also harbored a *divIVA* deletion and monitored asymmetric septation by fluorescence microscopy. Since *sigF* deletion results in a disporic phenotype in which two polar septa are elaborated, we grouped cells containing either one or two polar septa together in one category as being able to form at least one polar septum. At 1.5 hours after the induction of sporulation, wild type cells elaborated a polar septum in 67% of cells (n = 100; Fig. S6B); in the absence of σ^F^, 51% of the cells were still able to elaborate at least one polar septum at this time point (n = 100; Fig. S6C). In a Δ*minCD* Δ*sigF* strain 46% of the cells (n = 100; Fig. S6D) were able to form polar septa, a similar fraction as the Δ*sigF* strain (minicells were distinguished from forespores by observing the presence of DNA in forespores using DNA stain). In contrast, in the absence of DivIVA and σ^F^ there were no observable polar septa, and in a Δ*minCD* Δ*divIVA* Δ*sigF* strain only 7% of the cells displayed asymmetric septation (Fig. S6E–F; n = 100), suggesting that deletion of Δ*sigF* was not able to suppress the polar septation defect caused by the absence of DivIVA. We therefore conclude that DivIVA is required for the forespore-specific activation of σ^F^, and that the defect in polar septation observed in the absence of DivIVA is not due to the promiscuous activation of σ^F^ in a compartment-unspecific manner.

### Super-resolution microscopy reveals that DivIVA and SpoIIE persist on the forespore-side of the asymmetric septum

The biochemical basis of how SpoIIE activates σ^F^ has been extensively studied [23], [35]–[37], but the cell biological mechanism underlying how it exerts this function in a forespore-specific manner has remained largely unclear. Recently, Guberman et al. developed an algorithm which refines diffraction-limited images by interpolating the space between adjoining pixels and concluded that SpoIIE preferentially localizes to the forespore side of mature polar septa. To more directly visualize the localization of SpoIIE and DivIVA both at mature polar septa, and those septa that are just beginning to form, we employed a super-resolution fluorescence microscopy technique called Structured Illumination (SIM), which can potentially increase the resolution of fluorescence microscopy two-fold [63]. Indeed, this increase in resolution has previously allowed us to distinguish the localization of DivIVA-GFP on either side of a ∼80 nm wide division septum in vegetative cells [40].

**Figure 6:**
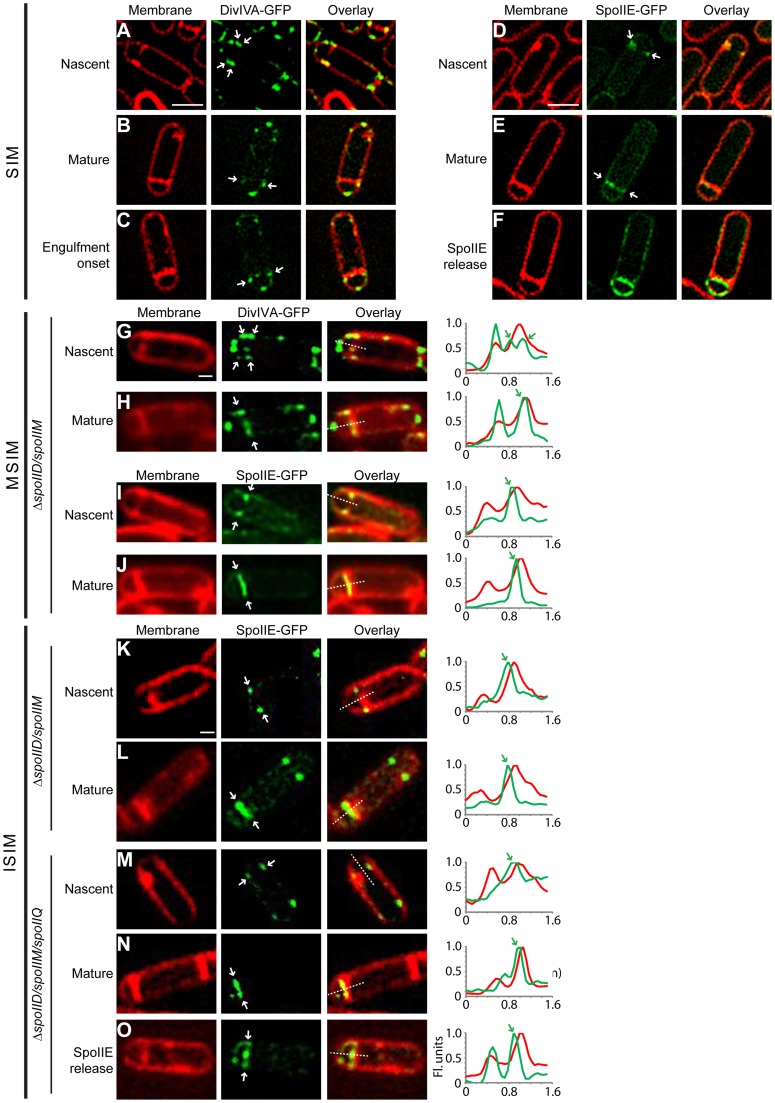
DivIVA and SpoIIE preferentially localize to the forespore side of the polar septum. Subcellular localization of DivIVA-GFP or SpoIIE-GFP was monitored using (A–F) structured illumination microscopy (SIM), (G–J) multifocal structured illumination microscopy (MSIM), or (K–O) instant structured illumination microscopy (ISIM). (A–C) Localization of DivIVA-GFP in sporulating cells (strain KR604) displaying (A) nascent, (B) mature, or (C) slightly curved polar septa, using SIM. (D–F) Localization of SpoIIE-GFP in sporulating cells (strain PE118) displaying (D) nascent or (E) mature septa. (F) Localization of SpoIIE after release into the membrane surrounding the forespore [29], using SIM. Localization of DivIVA-GFP in sporulating Δ*spoIID* Δ*spoIIM* cells (strain PE275) displaying (G) nascent, or (H) mature polar septa using MSIM. Localization of SpoIIE-GFP in sporulating Δ*spoIID* Δ*spoIIM* cells (strain PE274) displaying (I) nascent, or (J) mature polar septa using MSIM. Localization of SpoIIE-GFP in sporulating Δ*spoIID* Δ*spoIIM* cells (strain PE274) displaying (K) nascent, or (L) mature polar septa using ISIM. Localization of SpoIIE-GFP in sporulating Δ*spoIID* Δ*spoIIM* Δ*spoIIQ* cells (strain PE368) displaying (M) nascent, (N) mature polar septa using ISIM. (O) Localization of SpoIIE after release into the membrane surrounding the forespore [29], using ISIM. All white arrows indicate the localization of DivIVA-GFP or SpoIIE-GFP relative to the polar septum. Linescan analyses of normalized fluorescence intensity along the axis of the dashed line (along the entire width of the bacterium) in both channels at the selected polar septa are shown at the right; green arrows indicate the fluorescence from the GFP signal indicated with white arrows in the micrographs. Scale bars: (A–F), 1 µm; (G–O), 0.5 µm.

We first observed the localization pattern of DivIVA-GFP and SpoIIE-GFP using a commercially available SIM setup (DeltaVision OMX). As shown in Fig. 6A, membrane invagination at the onset of asymmetric division could be visualized by the increase in membrane staining near a pole along the lateral edges of the cell. DivIVA-GFP, produced under the control of its native promoter, localized initially on either side of this site of membrane invagination, similar to its reported behavior at nascent vegetative septa [40]. However, shortly after the completion of polar septation, DivIVA-GFP was found preferentially on the forespore side of the septum- a preference that persisted even after the onset of engulfment when the polar septum began to curve (Fig. 6B–C). This localization pattern was unlike the localization displayed by DivIVA-GFP during vegetative division, where DivIVA-GFP localized on both sides of a division septum and persisted on both sides long after septation was completed. The mean fluorescence intensity of polar DivIVA patches in cells harboring an adjacent polar septum (8286±3321, n = 50) was similar to that of cells harboring no adjacent septa (9046±3072, n = 50), consistent with a scenario in which no significant net exchange of DivIVA molecules appears to take place from the pole to the polar septum (compare patches in cell 1 and cell 2 in Fig. 1C).

We next observed the localization of SpoIIE-GFP under the control of its endogenous promoter in sporulating cells using the same SIM setup. At nascent polar septa, we observed that in many cells SpoIIE-GFP preferentially localized on the face of the polar septum that abutted the forespore (Fig. 6D). After septum formation was completed (Fig. 6E), SpoIIE localized to the forespore side of the septum, and finally, as reported previously, SpoIIE was released from the polar septum and localized uniformly in the membrane surrounding the forespore (Fig. 6F; [29]).

We were initially unable to properly quantify a large enough dataset of cells displaying the forespore-preferential localization of SpoIIE due to a laser-induced phototoxicity effect, using several different commercially available SIM setups, which deformed many of the polar septa that we observed (examples are shown in Fig. S7A). Curiously, we had not observed such severe phototoxic effects when examining vegetative septa. We therefore used a different implementation of this super-resolution technique called MSIM [64] that greatly reduced this phototoxic effect. To observe initial SpoIIE-GFP localization in a larger number of cells, we used a previously described engulfment-deficient strain (Δ*spoIIDM*) in which the polar septum remains flat [24]. Using MSIM, we again observed that DivIVA-GFP at nascent asymmetric septa localized initially on both forespore and mother cell sides (Fig. 6G). After polar septation was complete, in 81% of sporulating cells (n = 22), DivIVA-GFP persisted only on the forespore side of the mature septum (Fig. 6H), whereas in only 14% of cells, DivIVA-GFP localized on both sides of the polar septum.

In the case of SpoIIE-GFP, MSIM revealed that SpoIIE preferentially localized on the forespore side in both nascent and mature asymmetric septa (Fig. 6I–J; Fig. S9)- the shift in the GFP channel towards the forespore side was revealed more readily by the linescan graph of normalized fluorescence intensity of both membrane stain and SpoIIE-GFP at the septum. In total, 58% displayed clear forespore side specific SpoIIE-GFP localization (n = 38), while the remaining 42% displayed ambiguous localization in which the GFP channel largely overlapped that of the membrane stain. Of note, we did not detect any cells in which localization of SpoIIE-GFP was preferentially on the mother cell side of the polar septum.

One complication of this analysis, though, was that we were unable to determine if the forespore-proximal localization of SpoIIE-GFP in the cells occurred immediately upon septation or if we were imaging the cells after SpoIIE-GFP was released into the forespore membrane and subsequently recaptured. In order to eliminate from our analysis those cells that had recaptured SpoIIE-GFP, we examined SpoIIE-GFP localization in the absence of SpoIIQ, the protein responsible for the recapture of SpoIIE to the polar septum [29]. Whereas SpoIIE-GFP localized exclusively to the flat polar septum in 81% of engulfment-defective (Δ*spoIIDM*) sporulating cells (n = 100), in the absence of SpoIIQ, SpoIIE-GFP localized to the polar septum in only 52% of the sporulating cells (n = 118). In the remaining cells, SpoIIE-GFP was uniformly distributed around the forespore membrane, consistent with the inability of these cells to recapture SpoIIE at the polar septum ([29] (Fig. S8)). To verify on which face of the polar septum SpoIIE localized to initially before release into the forespore membrane, we employed yet another implementation of SIM, termed instant structured illumination microscopy (ISIM). This technique eliminates the need for the digital post-processing required in other SIM implementations, directly providing a ∼1.4-fold increase in resolution in the raw images, followed by subsequent deconvolution which then provides the full 2-fold resolution improvement relative to conventional widefield fluorescence imaging, allowing us to collect super-resolution images more rapidly [65]. Similar to the MSIM results, when using ISIM, DivIVA-GFP initially localized to both sides of the polar septum in 67% (n = 36) of engulfment-defective (Δ*spoIIDM*) sporulating cells (Fig. S10), while the remaining 33% displayed forespore-proximal localization. However, 76% of mature polar septa displayed DivIVA-GFP on the forespore-proximal side, while in the the remaining 24%, DivIVA-GFP was found on both sides (Fig. S10). Next, we examined SpoIIE-GFP localization using ISIM. SpoIIE-GFP appeared to localize preferentially on the forespore-side of the asymmetric septum (Fig. 6K–L) in engulfment-deficient (Δ*spoIIDM*) cells. In cells that additionally harbored a deletion of *spoIIQ*, in those cells which did not release SpoIIE-GFP into the forespore membrane, we observed that SpoIIE-GFP localized preferentially to the forespore-side in 64% (n = 45) of nascent polar septa and 67% (n = 69) of mature polar septa (Fig. 6M–O; Fig. S9), consistent with a model in which SpoIIE preferentially localizes to the forespore-proximal face of the polar septum before being released into the forespore membrane.

To further ensure that the forespore-biased localization of DivIVA-GFP and SpoIIE-GFP were not due to erroneous image registration by the image processing software, or due to variations in the microscope stage when the positions of emission filters changed, we conducted the experiments with fluorescent beads that fluoresce at both red and green wavelengths and aligned the beads' fluorescence in both channels as an internal control. In the example in Fig. S7B, the arrowhead indicates the location of a bead adjacent to a sporulating cell containing a nascent asymmetric septum. As shown in the overlay, there was no shift between red and green channel for the bead, whereas SpoIIE-GFP in the sporulating cell lying adjacent to the bead was preferentially localized to the forespore-proximal side of the polar septum (Fig. S7B). We conclude that DivIVA initially localizes to either side of nascent polar septa and localizes preferentially to the forespore side once septation is complete. SpoIIE, however, preferentially localizes to the forespore-proximal side of the polar septum from the very onset of membrane invagination and remains associated with the forespore-proximal face of the polar septum until it is released into the membranes surrounding the forespore.

## Discussion

DivIVA of *B. subtilis* is an extensively studied protein made of coiled-coil domains that resembles eukaryotic tropomyosins [53], [66], and has two well known functions. First, during vegetative growth, DivIVA arrives at mid-cell shortly after cytokinesis initiates and recruits the components of the Min system to mid-cell to prevent aberrant septation on either side of the site of membrane constriction [40], [44], [45], [47]. It has been proposed that an increase in negative membrane curvature, which arises on both sides of the division septum as the membrane constricts, drives this localization of DivIVA to mid-cell [41], [42]. Second, during sporulation, DivIVA localizes to the extreme cell poles and anchors the two origins of replication of the replicated chromosomes to each pole so that the forespore and mother cell each ultimately receive one copy of the chromosome [48]–[50]. In this report, we demonstrate a third role for DivIVA in which it localizes to the polar septum during sporulation and resides in complex with a multifunctional transmembrane protein called SpoIIE. SpoIIE is initially recruited to mid-cell at the onset of sporulation by FtsZ, after which it is required for the efficient redeployment of the Z-ring from mid-cell to polar positions to initiate asymmetric division. After asymmetric division commences, SpoIIE is initially recruited to the polar septum by FtsZ, but a significant population of SpoIIE remains associated with the polar septum even after FtsZ constricts and leaves that location. Shortly thereafter, SpoIIE is released exclusively into the forespore membrane to perform its second function in activating the first forespore-specific transcription factor σ^F^ [23], [30]. Subsequently, SpoIIE is recaptured at the forespore face of the polar septum [29] where it may participate in remodeling the peptidoglycan at the polar septum [22]. In the absence of DivIVA, we found that the two main functions of SpoIIE were disrupted, in that asymmetric septation did not occur, and that σ^F^ was prematurely activated in a compartment-unspecific manner. Interestingly, the deletion of several other cell division genes have only been reported to cause an asymmetric cell division defect, but not the premature activation of σ^F^ [58]–[61]. We therefore propose that, in addition to SpoIIE, DivIVA is required for the redeployment of FtsZ to the polar division sites and for the compartment-specific activation of σ^F^ in the forespore.

Our studies began with the observation that DivIVA localizes to the polar septum during sporulation. In an effort to determine the function of DivIVA at this location, we deleted *divIVA* and suppressed the elongation defect caused by this deletion by introducing a deletion in *minCD* as well. As previously observed, deletion of *minCD* alone had a mild defect in asymmetric division [67]–[70], but the additional deletion of *divIVA* resulted in the nearly complete abrogation of asymmetric division. That this defect was not primarily dependent on the absence of MinCD was established in an experiment in which we achieved the controlled degradation of DivIVA alone at a time point after it had completed its chromosome anchoring function, but before cells had initiated asymmetric division. Removal of DivIVA alone in this manner similarly abrogated asymmetric division at the onset of sporulation. Ultimately, we observed that the defect in asymmetric septation in the absence of DivIVA was due to the inability of these cells to redeploy FtsZ and SpoIIE from medial to polar positions. Interestingly, although polar septation was not directly measured at the time, Cha and Stewart had hypothesized that DivIVA may play an active role in the asymmetric positioning of the polar septation in the very first report of the *divIVA* locus in *B. subtilis* [68]. Additionally, a recent report suggested that a severe sporulation defect in a strain in which Spo0A rapidly accumulated at the onset of sporulation was perhaps due to the premature suppression of *divIVA* expression, consistent with our hypothesis that DivIVA plays an additional, indispensible role during sporulation [71]. Previously, Ben-Yehuda and Losick had demonstrated that, in vegetatively growing *B. subtilis*, the slight overproduction of FtsZ and the production of SpoIIE were sufficient to generate polar septa [18]. We propose that, in this context, DivIVA, which is abundant during vegetative growth, is also required for the redeployment of FtsZ to generate polar septa during vegetative growth.

It is currently unclear to us how DivIVA mechanistically mediates asymmetric division. At the onset of sporulation, the replicated chromosomes are extensively remodeled so that it may form the “axial filament” structure that extends from one pole to the other in what has traditionally been named “Stage I” of sporulation. Interestingly, in the absence of DivIVA, cells appeared to be arrested at this stage which, to our knowledge, is only the second description of a gene whose deletion results in a so-called “Stage I” arrest, not simply a delay [72]. Proper chromosome segregation has also been implicated in formation of the polar septum, in a manner that depends on the DNA-binding protein Spo0J [73]. Interestingly, DivIVA was shown to interact with Spo0J in a sporulation-specific manner, and independent of the Min system [74], [75]. Recently, the absence of RefZ, a DNA-binding protein that binds to origin- and terminus-proximal regions of the chromosome was reported to delay asymmetric septation, and it was proposed that RefZ may either facilitate repositioning of FtsZ at a polar position or promote disassembly of FtsZ at mid-cell sites [76]. It is therefore conceivable that the asymmetric septation impairment in the absence of DivIVA is a result of defective chromosome remodeling or segregation. It is also possible that the asymmetric septation defect in the absence of DivIVA may be through a metabolic pathway that affects septum formation. Deletion of *citC*, which encodes isocitrate dehydrogenase, also resulted in inhibition of asymmetric septation [72]. Loss-of-function mutations in the *spoVG* gene suppressed this defect, and overproduction of SpoVG in otherwise wild type cells resulted in delayed σ^F^ activation [77]. Curiously, SpoVG homologs were recently shown to be site-specific DNA-binding proteins [78], thereby possibly providing a link between asymmetric septation and chromosome remodeling or segregation.

**Figure 7:**
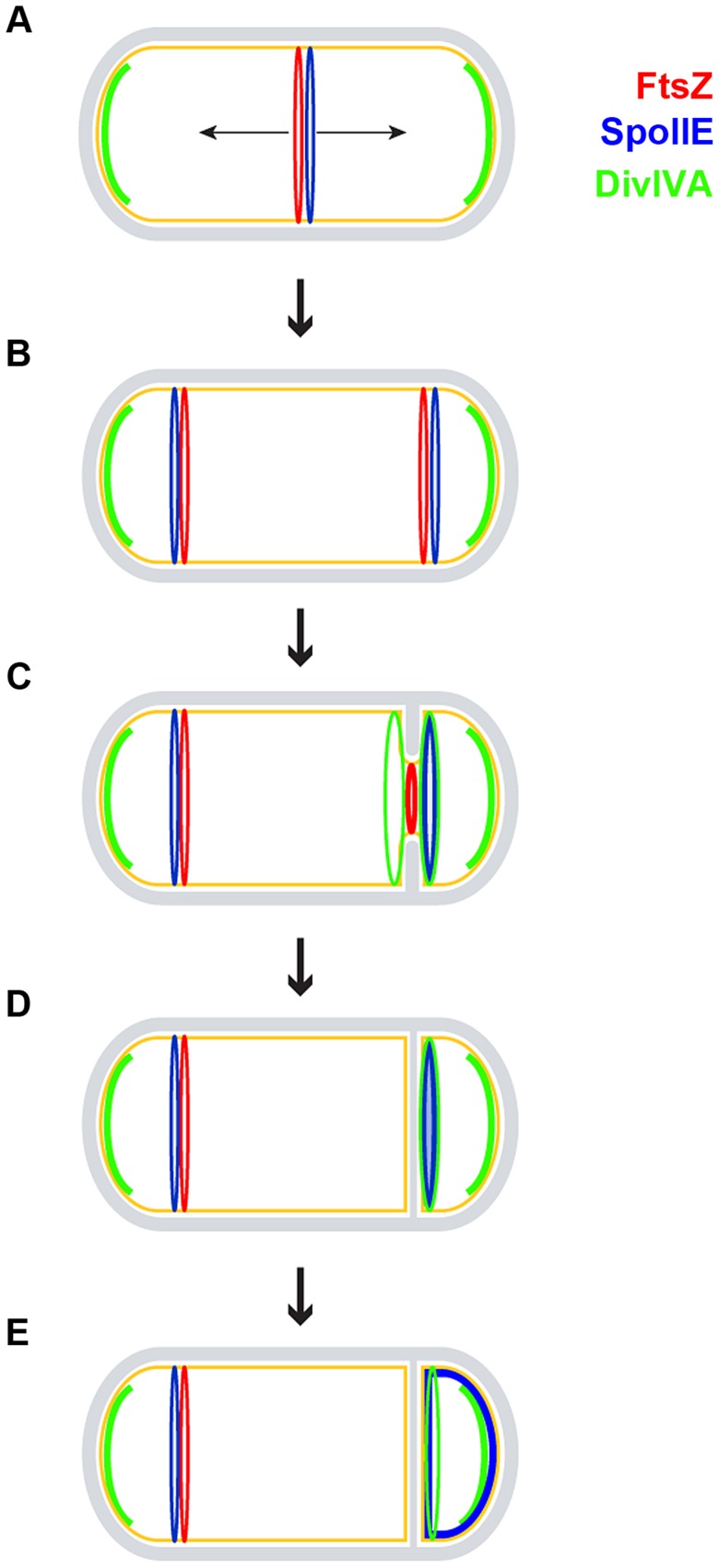
Model for the role of DivIVA during sporulation. A sporulating *B. subtilis* cell is depicted using the same color scheme as in Fig. 1A. (A) FtsZ (red ring) initially assembles in the pre-divisional cell at mid-cell and recruits SpoIIE (blue ring); at this time, DivIVA (green arcs) pre-localizes as patches at the two cell poles to perform its role in chromosome anchoring. (B) FtsZ and SpoIIE redeploy to polar division sites. In addition to SpoIIE and the increased expression of *ftsZ*, this redeployment requires DivIVA in a manner that is independent of MinCD and RacA, but the molecular mechanism of which is unclear. (C) One of the polar FtsZ rings constricts, while the other is eventually disassembled. Sensing membrane invagination, DivIVA localizes to either side of the nascent asymmetric septum and then sequesters SpoIIE to the septum. Although DivIVA is initially on both sides of the forming polar septum, SpoIIE preferentially localizes to the forespore side. (D) Upon completion of polar septum formation, both DivIVA and SpoIIE persist preferentially on the forespore side of the polar septum. (E) SpoIIE is released into the forespore membrane. It is subsequently recaptured at the polar septum by a protein produced under the control of σ^F^ [29].

In addition to its less understood role in redeploying the Z-ring from mid-cell to polar sites, SpoIIE is required for the activation of σ^F^, the biochemical basis of which has been well studied [23], [30]–[32], [34]. However, the cell biological basis for the forespore-specific activation of σ^F^ has been less well understood for two reasons. First, although FtsZ is thought to recruit SpoIIE to the polar septum, it had been unclear how SpoIIE remains associated with the polar septum after FtsZ constricts at that site and ultimately dissipates from that location, or how FtsZ may selectively release SpoIIE into the forespore membrane upon completion of constriction. We have observed a previously unappreciated step in which SpoIIE forms a ring-like structure at the polar septum during FtsZ constriction, before the release of SpoIIE into the forespore membrane. Based on our observation that DivIVA and SpoIIE co-localize at the polar septum and the discovery that DivIVA and SpoIIE copurify with each other, we propose that DivIVA is required for this initial sequestering of SpoIIE at the polar septum (Fig. 7). Consistent with this model, SpoIIE, when produced in vegetative cells, stably associated with vegetative division septa only in the presence of DivIVA. In sporulating cells, in the absence of DivIVA, polar septa were not efficiently formed and σ^F^ was prematurely activated in a compartment-unspecific manner. Second, the mechanism by which SpoIIE exerts its activity specifically in the forespore was also unclear. SpoIIE is released into the forespore membrane and it is promptly recaptured, nearly quantitatively, at the polar septum, suggesting that SpoIIE is exclusively released into the forespore, and not into the mother cell membrane [29]. Another report suggested, based on the interpolation of fluorescence signal in diffraction-limited images, that at mature polar septa SpoIIE was preferentially detected on the forespore side of the polar septum [39] -this localization of SpoIIE was likely after its recapture at the polar septum. In this report, we examined cells that displayed nascent polar septa, as well as cells that had elaborated mature polar septa, using three super-resolution techniques called SIM, MSIM, and ISIM, and observed that the biased localization of SpoIIE on the forespore side could be observed at the very onset of membrane invagination. In contrast, we observed DivIVA initially on either side of nascent polar septa, similar to its localization pattern in vegetative division septa. However, unlike its behavior in vegetative cells where it was ultimately equally distributed on both sides, we observed that at mature polar septa, DivIVA, like SpoIIE, also preferentially localized to the forespore side. The mechanism for this shift in distribution is currently unclear. It may, for example, involve the selective degradation of DivIVA on the mother cell face of the polar septum or may involve a transfer of pole-localized DivIVA molecules to the forespore-proximal side of the polar septum which we were unable to measure using our current experimental setup. Such a transfer has indeed been recently reported for vegetative septa, which may occur in 5–20 mins [79]. Curiously, the presence of Min proteins, which are typically recruited by DivIVA, on the forespore-proximal face of the polar septum has been implicated in maintaining the polarity of DNA translocation [80]. The basis for the early bias in localization of SpoIIE is currently not known, but this pattern highlights the establishment of asymmetry, at a compartment-specific level, in the developing sporangium long before polar septation is completed. We propose that the interaction of SpoIIE with DivIVA allows for its retention at the forespore side of the newly forming polar septum until septation is complete. After completion of polar septum formation, similar to the release of MinCD after completion of vegetative septa [47], SpoIIE may be liberated to redistribute along the membrane surrounding the forespore. It is tempting to speculate that the initial interaction of SpoIIE with DivIVA may inhibit the phosphatase activity of SpoIIE until it is released into the forespore membrane, which may also explain the premature activation of σ^F^ in the predivisional cell in the absence of DivIVA. Curiously, the N-terminus of MinC shares structural similarity with that of SpoIIAA, the protein that is dephosphorylated by SpoIIE [81]. Thus, once released into the forespore membrane, the phosphatase activity of SpoIIE may be uninhibited and σ^F^ may be activated. Therefore, asymmetrically placed DivIVA may act as a molecular beacon that signals the completion of septum formation for the subsequent activation of σ^F^. An outstanding question, though, will be to determine how the asymmetry of SpoIIE is established at such an early time point before the mother cell and forespore compartments are even separated.

## Materials and Methods

### Strain construction and general methods

All strains used in this study are congenic derivatives of *B. subtilis* PY79 [82]. Genotypes of strains used are provided in Table S1. *B. subtilis* competent cells were prepared as described previously [83]. β-galactosidase activity of cell samples collected at time points indicated was measured with modifications as described previously [84], [85]. To express *divIVA-linker-cfp* from the *amy* locus, *P_divIVA_-divIVA* without the stop codon was PCR amplified from PY79 chromosomal DNA using primers that abutted *Hind*III and *Nhe*I sites (“oP10” 5′-AAAAAGCTTTCGTGTTTTCTGAGACA and “oP11” 5′-AAAGCTAGCTTCCTTTTCCTCAAATAC). *linker-cfp* was PCR amplified from DS4152 chromosomal DNA [44] using primers abutting *Nhe*I and *BamH*I sites (“oP54” 5′-AAAGCTAGCGGT TCCGCTGGCTCCGCTGCTGGT TCTGGCCTC and “oP55” 5′-AAAGGATCCTTACTTATAAAGTTCGTCCATGCCAAGTGTAATGCC). Both fragments ligated into integration vector pDG1662 to create pPE17. To express *divIVA-linker-gfp* from the *amy* locus, pPE17 was digested with *Hind*III and *Xho*I to liberate *P_divIVA_-divIVA* and part of the linker sequence. Next, *gfp mut2* was PCR amplified from pKC2 [86] using primers that abutted the remaining linker sequences, and 5′ *Xho*I and 3′ *BamH*I sites (“oP62” 5′-AAACTCGAGGGTTCCGGAATGAGTAAAGGAGAAGAACTTTTC and “oP47” 5′-AAAGGATCCTTATTTGTATAGTTCATCCATGCC). Both fragments were ligated into pDG1662 to create pKR227. To replace *divIVA* at the native locus with *divIVA-FLAG-ssrA^Ec^*, the final 400 nucleotides of *divIVA* (omitting the stop codon) were PCR amplified using primers that abutted *BamH*I and *Xba*I sites (“DivIVA-C4005′Bam” 5′-AAAGGATCCAGTCAGAAAAGATTACGAAATTG and “DivIVA-C4003′ Xba” 5′ AAATCTAGATTCCTTTTCCTCAAATACAGCG), and ligated into Campbell integration vector pKG1268 [52] to create pKR226. To express *divIVA-FLAG* from *amyE*, *P_divIVA_-divIVA* was PCR amplified from PY79 chromosomal DNA using primers that abutted 5′ *Hind*III and 3′ *BamH*I sites, as well as a 3′ FLAG tag sequence (“DivIVAprom5′Hind” 5′-CCCAAGCTTTCGTGTTTTCTGAGACAGCAG and “DivIVA3′FLAGBam” 5′-CGCGGATCCTTACTTGTCGTCATCGTCTTTGTAGTCTTCCTTTTCCTCAAATACAG), and ligated into pDG1662 to create pKR200. To express *spoIIE-gfp* from *amyE* under the control of an IPTG-inducible promoter, *spoIIE-gfp* was PCR amplified from SB201 [87] using primers that abutted *Sal*I and *Sph*I sites (“oP44” 5′- AAAGTCGACACATAAGGAGGAACTACTATGGAAAAAGCAGAAAGAAGAGTGAACGGG and “oP24” 5′- GCCGCATGCTTATTTGTATAGTTCATCCATGCC), and ligated into integration vector pDR111 to create pPE19. To express IPTG-inducible *spoIIE-FLAG* from *amyE*, plasmid pPE48 was created by appending 3×-flag tag sequence (GATTATAAGGATCATGATGGTGATTATAAGGATCATGATATCGACTACAAAGACGATGACGACAAG) followed by a stop codon to the 3′ end of the *spoIIE* coding sequence in pPE19 via QuikChange mutagenesis (Agilent). Strain KR610 (Δ*spoIIE::tet*) was created by the long flanking homology method [88] using primers (“spoIIEKO-1” 5′-GCAAGTAGCCTTGTTGACAC, “spoIIEKO-2” 5′-CAATTCGCCCTATAGTGAGTCGTTCCTCTCATCTCCCACCTG, “spoIIEKO-3” 5′-CCAGCTTTTGTTCCCTTTAGTGAGCGCTTCCGTATAAATCAAATTTC, and “spoIIEKO-4” 5′-TTTCAAGACATTCACTTCAGAAG). For immunoblot analysis, cells were grown as described below, harvested, and cell extracts for immunoblot analysis were prepared by lysozyme treatment as described previously [89]. Extracts were separated by SDS-PAGE and immunoblotted using antisera raised against purified DivIVA-GFP, σ^A^, GFP (Covance, Inc.), or *E. coli* FtsZ (courtesy of Sue Wickner; [90]) as indicated. Where indicated, band intensities were quantified using ImageQuant software (GE).

### Media and culture conditions

*B. subtilis* overnight cultures grown at 22°C in casein hydrolysate (CH) medium were diluted 1∶20 into fresh CH medium and grown until OD_600_ reached ∼0.5 at 37°C. For induction of sporulation, cells were spun down and resuspended in Sterlini and Mandelstam (SM) medium as described previously [91], for the time indicated. Sporulation efficiency was measured by growing cells in Difco sporulation medium (DSM; KD Medical) for at least 24 h at 37°C. The number of heat-resistant colony forming units (cfu) was obtained after incubation at 80°C for 10 min. For strains harboring genes that disrupted the *thr* locus, L-threonine (40 µg/ml final concentration) was added to the culture as a nutritional supplement. Where specified, IPTG (1 mM final concentration) was added at the indicated time to induce genes; xylose (0.5% final concentration), where indicated, was added at the time of resuspension.

### Epifluorescence microscopy

Cells were visualized as described previously [92]. Briefly, culture pellets (from 1 mL culture) that were grown as described above was washed with PBS and resuspened in 50–100 µL PBS containing 1 µg/ml (final concentration) of the fluorescent dye FM4-64 or 46 µg/ml (final concentration) TMA-DPH to visualize membranes and, where indicated, 2 µg/ml (final concentration) of DAPI to visualize DNA. Cells (5 µl) were then placed on a poly-L-lysine coated glass bottom culture dish (Mattek Corp.; poly-L-lysine did not appreciably affect localization of DivIVA-GFP at the polar septum (Fig. S2A)). A pad made of 1% agarose in distilled water (or SM media containing IPTG and FM4-64 for time lapse microscopy) was cut to size and placed above the cells. Cells were viewed at room temperature (or 32°C for timelapse) with a DeltaVision Core microscope system (Applied Precision) equipped with a Photometrics CoolSnap HQ2 camera and an environmental chamber. Seventeen planes were acquired every 200 nm at room temperature and the data were deconvolved using SoftWorx software. Imaris software was used for three dimensional surface rendering of fluorescence data. For 3D-SIM, cells were prepared as described above and imaged using Delta Vision OMX Blaze (Applied Precision). For MSIM, cells (with fluorescent beads when indicated) were labeled with FM4-64 as described above and placed on top of a glass slide and a freshly prepared poly-L-lysine coated coverslip (#1.5 thickness, VWR) was placed on top of the cell suspension; coverslips and slides were cleaned as described previously [93]. Fluorescence images were acquired using a custom MSIM system equipped with a 60× 1.45NA oil objective (Olympus) and appropriate filters (Chroma, zt405/488/561 (dichroic) LP02-488RE-25, NF03-561E-25 (emission filters). Total exposure times for each 2D slice were either 1 s or 2 s depending on the signal intensity. 2D slices were acquired every 200 nm along the Z-axis for construction of 3D volumes, comprising a total axial range of 10–12 µm for each sample. The longer wavelength channel (red) was collected prior to the shorter wavelength channel (green) for all samples. Multicolor 100 nm diameter Tetraspeck microspheres (Life Technologies, T-7279) were immobilized on the coverslip surface to enable precise alignment of each image channel. Alignment of the two channels was completed by translating the red channel relative to the green channel to maximize overlap of the reference microspheres using ImageJ. Images were deconvolved with a 3D Richardson-Lucy algorithm implemented in the python programming language (40 iterations, using a Gaussian PSF with x, y, z FWHM values 3.8×3.8×2.4 pixels, code available at code.google.com/p/msim) [64]. ISIM imaging was performed using a previously described system [65]. Cells were prepared as described above. Fluorescence from labeled membranes was excited at 561 nm and GFP signal from fusion proteins was excited at 488 nm. A 525 nm bandpass filter (Semrock, FF01-525/50-25) was used for GFP collection and a 561 nm notch filter (Semrock, NF03-561E-25) was used for collecting membrane fluorescence. Exposure times for imaging the membrane probe were 40 ms or 80 ms (depending on sample brightness) and 400 ms for imaging GFP fusion constructs. 3D stacks were collected with a z step size of 200 nm. Resulting images were deconvolved using Richardson-Lucy deconvolution, and image channels were aligned using the multicolor beads for reference as described above. Line scans were completed using the line scan function in ImageJ, setting the line width to 5-pixels. Line scan measurements were exported to Microsoft Excel for rendering and display.

### Coimmunoprecipitation

FLAG-tagged proteins were immunoprecipitated from strains coproducing FLAG- and GFP-tagged proteins using the FLAG Immunoprecipitation kit (Sigma). Samples were processed largely as described previously [84]. Briefly, a 20 ml culture of cells was induced to sporulate for 1.5 h, harvested, resuspended in 500 µl protoplast buffer (0.5 M sucrose, 20 mM MgCl_2_, 10 mM potassium phosphate [pH 6.8], 0.1 mg/ml lysozyme), and incubated at 37°C for 15 min to remove cell wall [94]. Protoplasts were harvested and cell extracts were prepared by resuspension in 1 ml of lysis buffer (50 mM Tris [pH 7.4], 150 mM NaCl, 1 mM EDTA, 1% Triton X-100). 100 µl of the extract was retained for analysis as the “load” fraction. The remaining cell extract was added to 20 µl of anti-FLAG affinity resin and incubated overnight at 4°C with light shaking. The antibody resin was centrifuged and supernatant was collected as the “unbound” fraction. The resin was then washed extensively with lysis buffer. Proteins associated with the resin were eluted through competitive elution with FLAG peptide-containing lysis buffer. Fractions were analyzed by immunoblotting using specific antisera as described above.

## Supporting Information

Figure S1 DivIVA-FLAG, DivIVA-FLAG-SsrA^Ec^, DivIVA-linker-GFP, SpoIIE-GFP, and SpoIIE-FLAG are largely functional in vivo. (A) Histogram displaying the individual cell lengths of various *B. subtilis* strains as measured by fluorescence microscopy of cells labeled with the membrane dye FM4-64: WT (strain PY79), Δ*divIVA* (strain KR546), or cells producing DivIVA-FLAG (strain KR557), DivIVA-FLAG-SsrA^Ec^ (strain KR600), or DivIVA-linker-GFP (KR606) as the only copy of DivIVA. Individual measurements for 100 different cells are displayed for each strain. (B) Mean cell size for the strains described in (A). Error bars represent standard deviation from the mean. (C) Fraction of minicells produced by strains harboring various alleles of *divIVA* as measured by fluorescence microscopy of cells labeled with FM4-64. First column: strains as described in (A); second column: total number of normal or elongated cells enumerated; third column: total number of minicells enumerated; fourth column: percent of total cells in the population that were minicells. (D) Sporulation efficiencies of strains harboring various alleles of *spoIIE* as measured by heat resistance (relative to WT). First column: WT (strain PY79), Δ*spoIIE* (strain KR610), *spoIIE-GFP* (strain PE180), *spoIIE-FLAG* (strain PE390) as the only copy of *spoIIE*. Strain PY79 produced 7.4×10^7^ (±1.7×10^7^) spores/ml; std dev, standard deviation from mean from three independent trials.
(TIF)

Figure S2 Localization of DivIVA-GFP at the polar septum. (A) Localization of DivIVA-GFP (strain KR604) using coverslips treated (top) or untreated (bottom) with poly-L-lysine to immobilize the cells. Scale bar: 2 µm. (B) Timelapse images of a sporulating *B. subtilis* cell producing DivIVA-GFP (strain KR541) while elaborating a polar septum. Arrows indicate polar septa; time (min) is indicated on the left.
(TIF)

Figure S3 Degradation of DivIVA-SsrA^Ec^ by IPTG-induced production of SspB. Immunoblot analysis of cells induced to sporulate and harvested at the times indicated above, producing DivIVA-SsrA^Ec^ (left; strain PE304), or DivIVA-SsrA^Ec^ and SspB (strain PE330) in the absence (center) or presence (right) of IPTG added at 45 min to induce expression of *sspB*, using antisera specific to DivIVA or σ^A^.
(TIF)

Figure S4 FtsZ protein levels and use of ZapA-GFP as a proxy for FtsZ localization. (A) Immunoblot analysis, using antisera specific to FtsZ or σ^A^, of *B. subtilis* cell extracts prepared at the times indicated (h) after the induction of sporulation. Shown are three independent trials (numbered on the right) from independent sporulating cultures of the following *B. subtilis* strains: WT (PY79); Δ*spo0A* (PE362); Δ*minCD* (KR620); Δ*divIVA* (KR543);Δ*minCD* Δ*divIVA* (PE308). (B) Localization of ZapA-GFP (top; strain PE290) in cells either 60 min or 120 min after the induction of sporulation, as indicated. Arrows indicate ZapA-GFP signal at polar division sites.
(TIF)

Figure S5 SpoIIE-GFP is solubilized by the nonionic detergent Triton X-100. Immunoblot analysis, using antisera specific to GFP, DivIVA, or σ^A^, of *B. subtilis* cell extracts (strain PE130), which overproduces SpoIIE-GFP, prepared 1.5 h after the induction of sporulation and separated into soluble supernatant (S) and insoluble pellet (P) fractions either without (−TX-100) or with (+TX-100) extraction with the nonionic detergent Triton X-100 in lysis buffer (see Materials and Methods for buffer components). Asterisk indicates a soluble GFP-tagged species that is likely a truncated form of SpoIIE-GFP.
(TIF)

Figure S6 Premature activation of σ^F^ is not responsible for the asymmetric septation defect in the absence of DivIVA. (A) β-galactosidase accumulation was measured at different time points after the induction of sporulation in cells harboring a σ^F^-dependent *P_spoIIQ_-lacZ* reporter fusion in otherwise wild type cells (•; strain PE300), Δ*minCD* (▪; strain PE321), Δ*divIVA* (▴; strain PE322), or Δ*divIVA* Δ*minCD* (▾; strain PE327). (B–F) Polar septum formation was monitored using the fluorescent membrane dye FM4-64 in cells that had initiated sporulation for 2 h in (B) wild type cells (strain PE80), (C) Δ*sigF* (strain RL1275); (D) Δ*sigF* Δ*minCD* (strain PE196); (E) Δ*sigF* Δ*divIVA* (strain PE199); (F) Δ*sigF* Δ*minCD* Δ*divIVA* (strain PE198). First panel: membranes visualized using FM4-64; second panel: chromosomes visualized using DAPI; third panel: overlay of membranes and DNA. Fraction of cells elaborating a polar septum is indicated to the right (ND, none detected).
(TIF)

Figure S7 Super-resolution micrographs of sporulating *B. subtilis* cells. (A) Examples of types of deformation to the polar septum that were routinely observed using the lowest laser power available when viewing the cells using several commercial SIM setups: DeltaVision OMX Blaze (top row), Nikon N-SIM (middle row), or Zeiss Elyra (bottom row) at either nascent (left column) or mature (right column) polar septa. Arrows indicate the site of deformation. (B) Localization of SpoIIE-GFP in sporulating Δ*spoIID* Δ*spoIIM* cells (strain PE274) observed using MSIM. Internal calibration of fluorescence from red and green channels using a bead that fluoresces in both channels (arrowhead) as viewed at (top) a plane close to the coverslip or at (bottom) an intermediate plane. Scale bar: 0.5 µm.
(TIF)

Figure S8 Localization of SpoIIE-GFP in the absence of SpoIIQ and engulfment. Subcellular localization of SpoIIE-GFP in mutant cells arrested at the flat septum stage before the onset of engulfment, 1.5 h after the induction of sporulation, in the presence (above, strain PE274) or absence (below, strain PE368) of SpoIIQ.
(TIF)

Figure S9 Gallery of sporulating cells displaying forespore-biased localization of SpoIIE-GFP. Subcellular localization of SpoIIE-GFP in mutant cells arrested at the flat septum stage before the onset of engulfment, 1.5 h after the induction of sporulation, in the presence (left, strain PE274) or absence (right, strain PE368) of SpoIIQ, visualized using either the MSIM or ISIM super-resolution technique. Arrows indicate fluorescent beads that were visible at mid-plane used for image registry.
(TIF)

Figure S10 Preferential localization of DivIVA-GFP to the forespore side of the polar septum. Localization of DivIVA-GFP in sporulating Δ*spoIID* Δ*spoIIM* cells (strain PE275) displaying (A) nascent or (B) mature polar septa visualized using ISIM. Arrowhead indicates a fluorescent bead used for image registry that was visible at mid-plane. Linescan analyses of normalized fluorescence intensity along the axis of the dashed line in both channels at the selected polar septa are shown at the right; green arrows indicate the fluorescence from the GFP signal indicated with white arrows in the micrographs. Scale bar: 0.5 µm.
(TIF)

Table S1 *Bacillus subtilis* strains used in this study.
(DOC)
