# Supplementary figures and images for: Asymmetric Division and Differential Gene Expression during a Bacterial Developmental Program Requires DivIVA
Source: PLoS Genet. 2014 Aug 7;10(8):e1004526. doi: 10.1371/journal.pgen.1004526 (PMC4125091; doi:10.1371/journal.pgen.1004526)

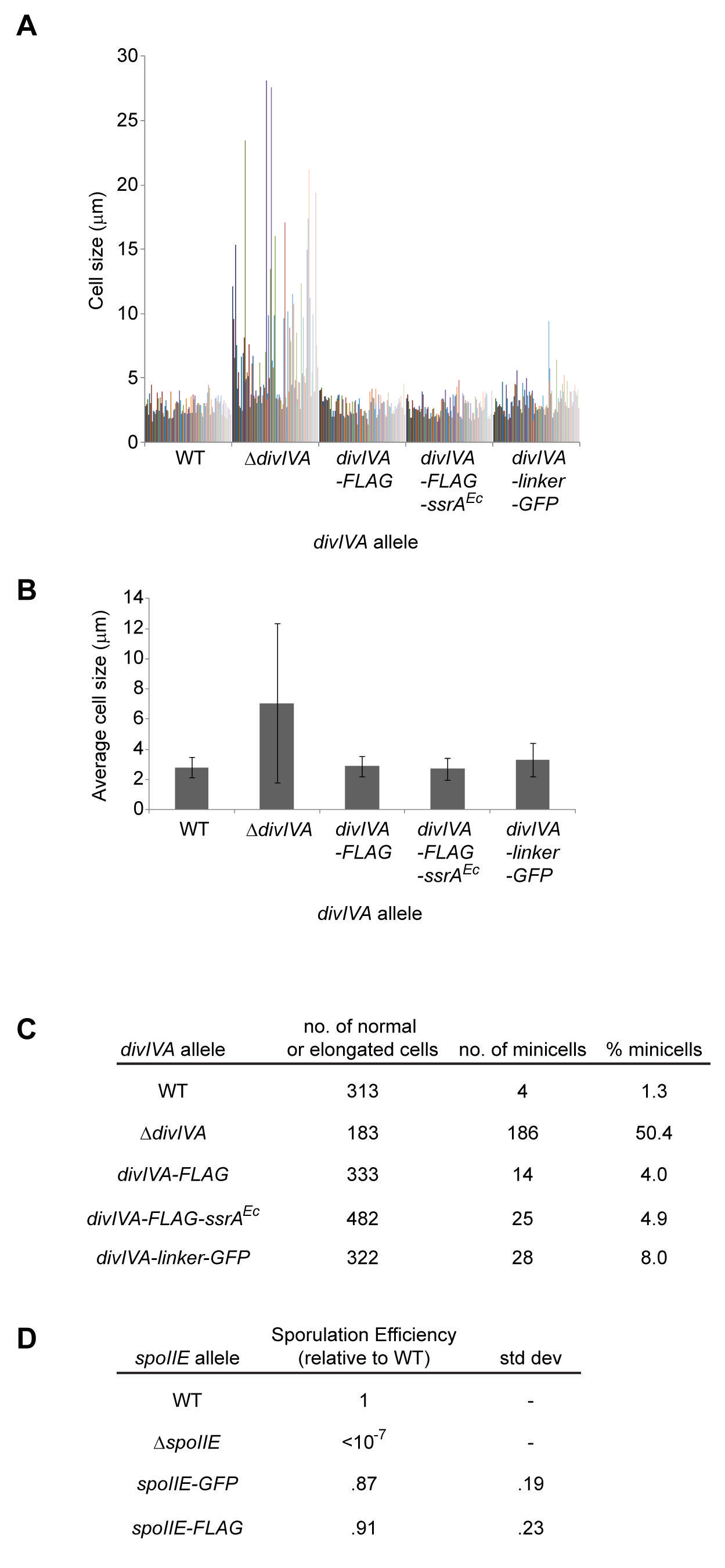

Supplement: Figure S1 — DivIVA-FLAG, DivIVA-FLAG-SsrAEc, DivIVA-linker-GFP, SpoIIE-GFP, and SpoIIE-FLAG are largely functional in vivo. (A) Histogram displaying the individual cell lengths of various B. subtilis strains as measured by fluorescence microscopy of cells labeled with the membrane dye FM4-64: WT (strain PY79), ΔdivIVA (strain KR546), or cells producing DivIVA-FLAG (strain KR557), DivIVA-FLAG-SsrAEc (strain KR600), or DivIVA-linker-GFP (KR606) as the only copy of DivIVA. Individual measurements for 100 different cells are displayed for each strain. (B) Mean cell size for the strains described in (A). Error bars represent standard deviation from the mean. (C) Fraction of minicells produced by strains harboring various alleles of divIVA as measured by fluorescence microscopy of cells labeled with FM4-64. First column: strains as described in (A); second column: total number of normal or elongated cells enumerated; third column: total number of minicells enumerated; fourth column: percent of total cells in the population that were minicells. (D) Sporulation efficiencies of strains harboring various alleles of spoIIE as measured by heat resistance (relative to WT). First column: WT (strain PY79), ΔspoIIE (strain KR610), spoIIE-GFP (strain PE180), spoIIE-FLAG (strain PE390) as the only copy of spoIIE. Strain PY79 produced 7.4×10^7^ (±1.7×10^7^) spores/ml; std dev, standard deviation from mean from three independent trials. (TIF) [file pgen.1004526.s001.tif]

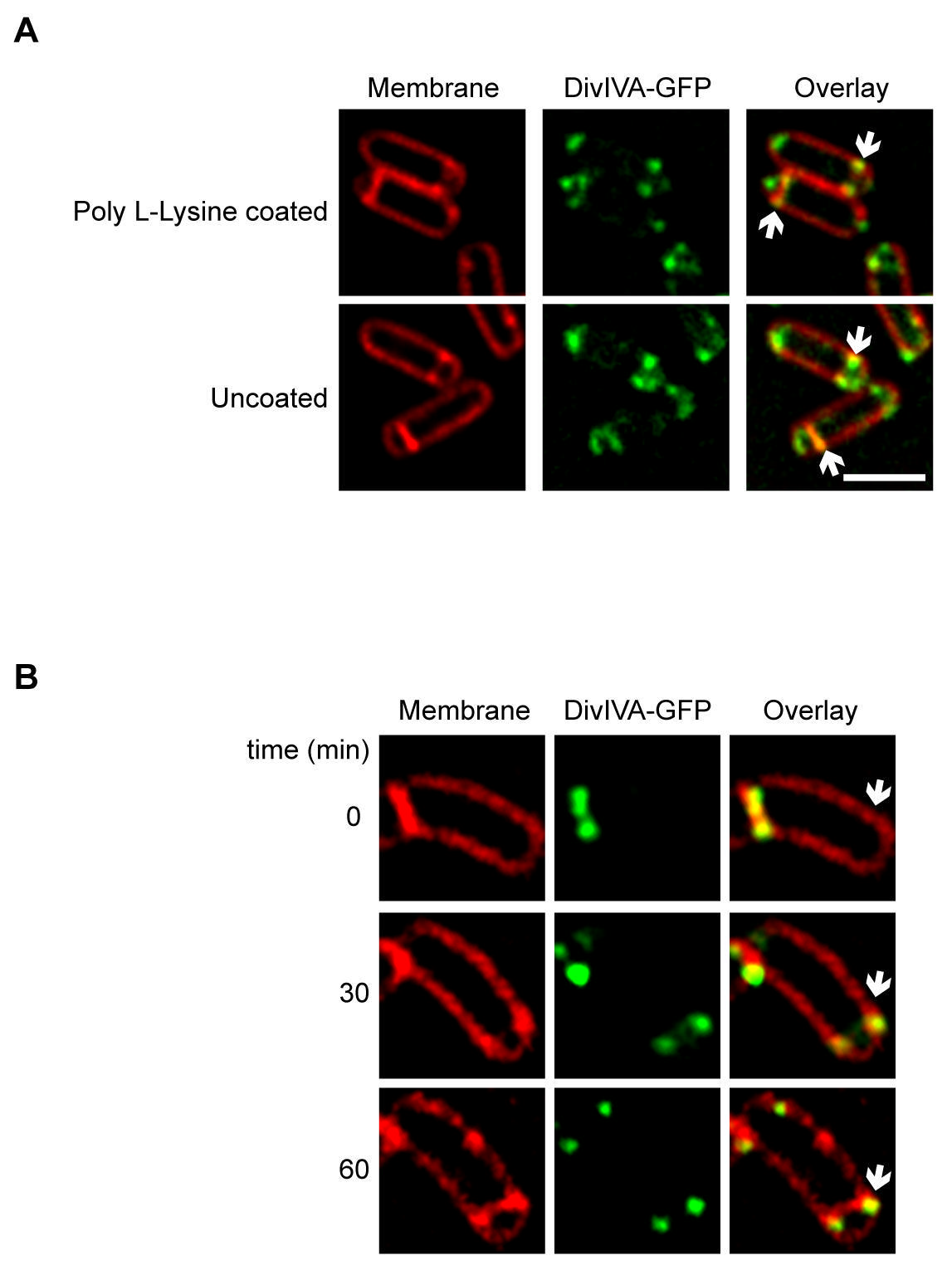

Supplement: Figure S2 — Localization of DivIVA-GFP at the polar septum. (A) Localization of DivIVA-GFP (strain KR604) using coverslips treated (top) or untreated (bottom) with poly-L-lysine to immobilize the cells. Scale bar: 2 µm. (B) Timelapse images of a sporulating B. subtilis cell producing DivIVA-GFP (strain KR541) while elaborating a polar septum. Arrows indicate polar septa; time (min) is indicated on the left. (TIF) [file pgen.1004526.s002.tif]

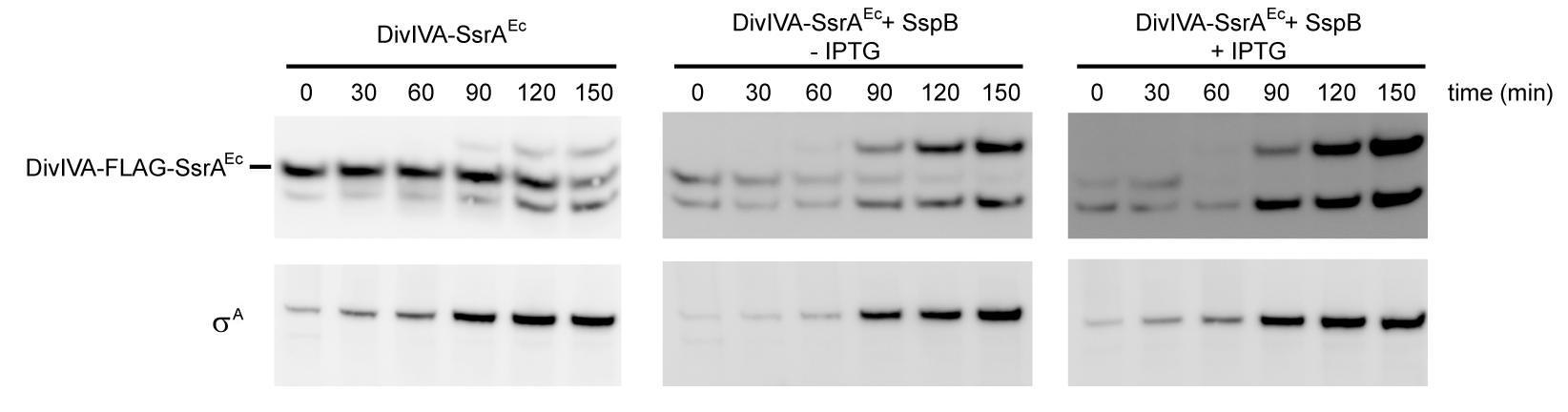

Supplement: Figure S3 — Degradation of DivIVA-SsrAEc by IPTG-induced production of SspB. Immunoblot analysis of cells induced to sporulate and harvested at the times indicated above, producing DivIVA-SsrAEc (left; strain PE304), or DivIVA-SsrAEc and SspB (strain PE330) in the absence (center) or presence (right) of IPTG added at 45 min to induce expression of sspB, using antisera specific to DivIVA or σA. (TIF) [file pgen.1004526.s003.tif]

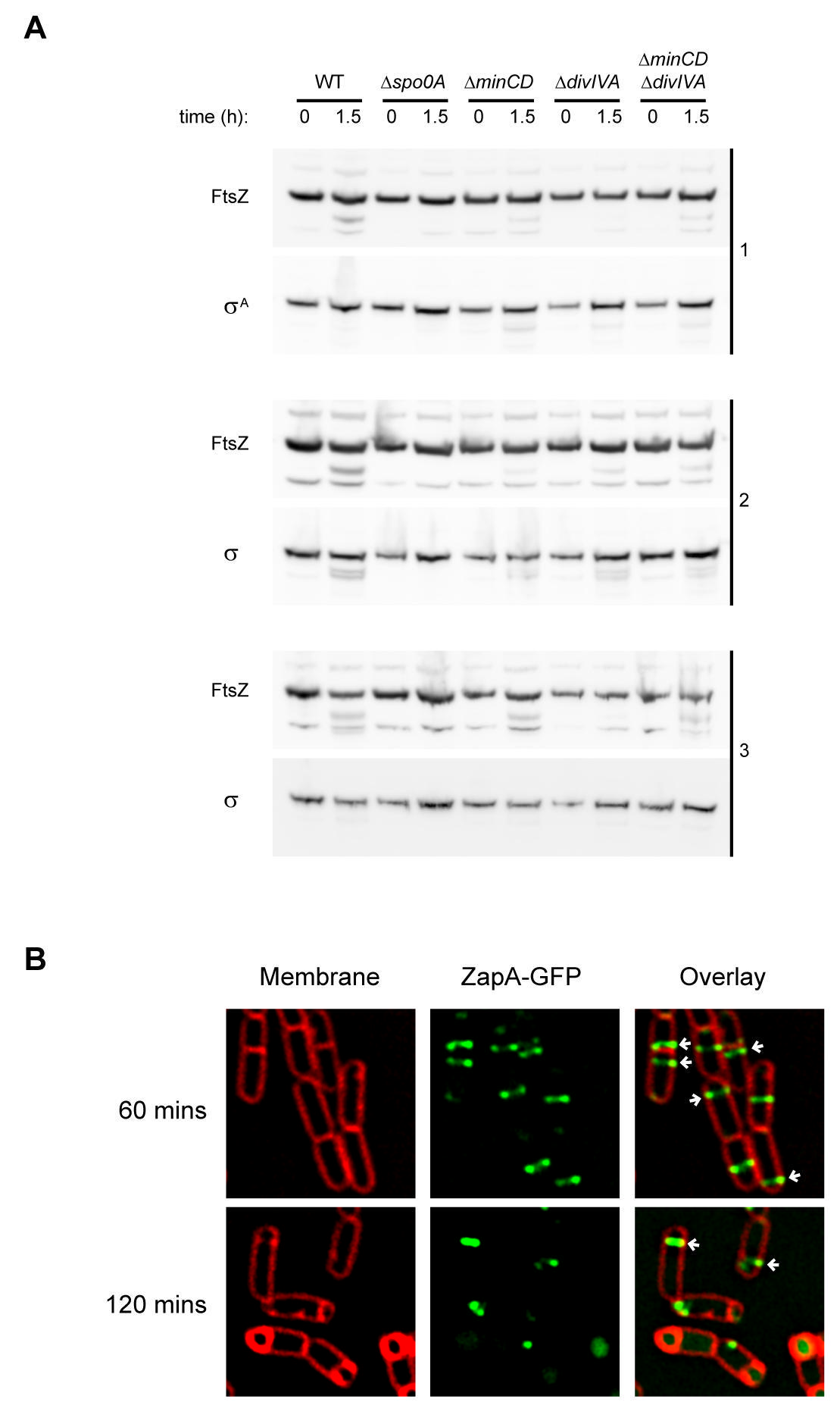

Supplement: Figure S4 — FtsZ protein levels and use of ZapA-GFP as a proxy for FtsZ localization. (A) Immunoblot analysis, using antisera specific to FtsZ or σA, of B. subtilis cell extracts prepared at the times indicated (h) after the induction of sporulation. Shown are three independent trials (numbered on the right) from independent sporulating cultures of the following B. subtilis strains: WT (PY79); Δspo0A (PE362); ΔminCD (KR620); ΔdivIVA (KR543);ΔminCD ΔdivIVA (PE308). (B) Localization of ZapA-GFP (top; strain PE290) in cells either 60 min or 120 min after the induction of sporulation, as indicated. Arrows indicate ZapA-GFP signal at polar division sites. (TIF) [file pgen.1004526.s004.tif]

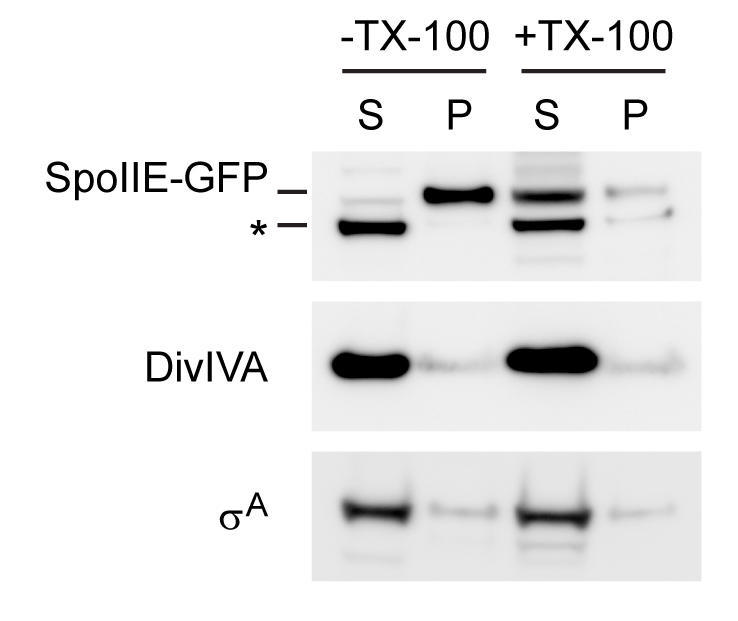

Supplement: Figure S5 — SpoIIE-GFP is solubilized by the nonionic detergent Triton X-100. Immunoblot analysis, using antisera specific to GFP, DivIVA, or σA, of B. subtilis cell extracts (strain PE130), which overproduces SpoIIE-GFP, prepared 1.5 h after the induction of sporulation and separated into soluble supernatant (S) and insoluble pellet (P) fractions either without (−TX-100) or with (+TX-100) extraction with the nonionic detergent Triton X-100 in lysis buffer (see Materials and Methods for buffer components). Asterisk indicates a soluble GFP-tagged species that is likely a truncated form of SpoIIE-GFP. (TIF) [file pgen.1004526.s005.tif]

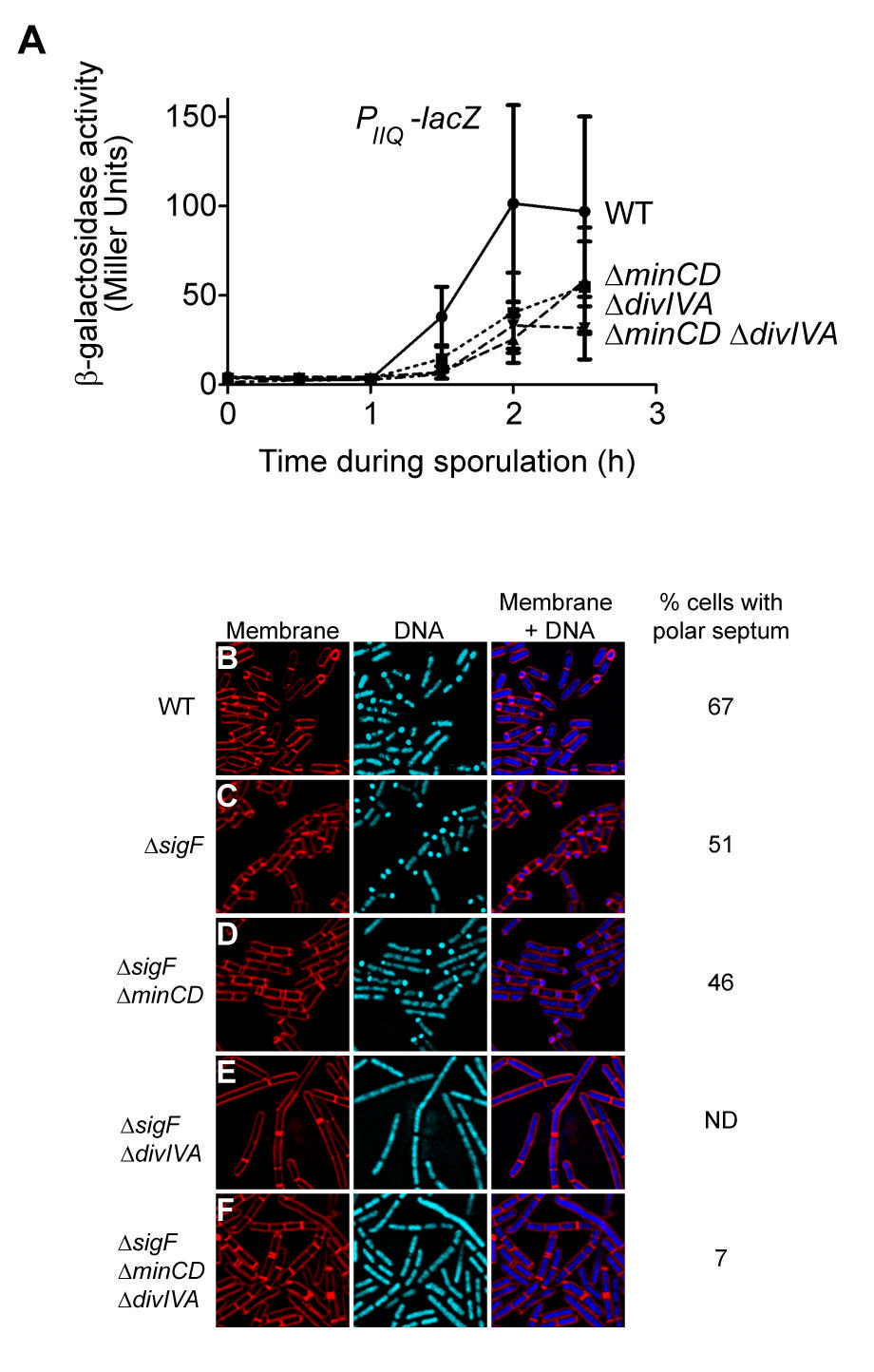

Supplement: Figure S6 — Premature activation of σF is not responsible for the asymmetric septation defect in the absence of DivIVA. (A) β-galactosidase accumulation was measured at different time points after the induction of sporulation in cells harboring a σF-dependent PspoIIQ-lacZ reporter fusion in otherwise wild type cells (•; strain PE300), ΔminCD (▪; strain PE321), ΔdivIVA (▴; strain PE322), or ΔdivIVA ΔminCD (▾; strain PE327). (B–F) Polar septum formation was monitored using the fluorescent membrane dye FM4-64 in cells that had initiated sporulation for 2 h in (B) wild type cells (strain PE80), (C) ΔsigF (strain RL1275); (D) ΔsigF ΔminCD (strain PE196); (E) ΔsigF ΔdivIVA (strain PE199); (F) ΔsigF ΔminCD ΔdivIVA (strain PE198). First panel: membranes visualized using FM4-64; second panel: chromosomes visualized using DAPI; third panel: overlay of membranes and DNA. Fraction of cells elaborating a polar septum is indicated to the right (ND, none detected). (TIF) [file pgen.1004526.s006.tif]

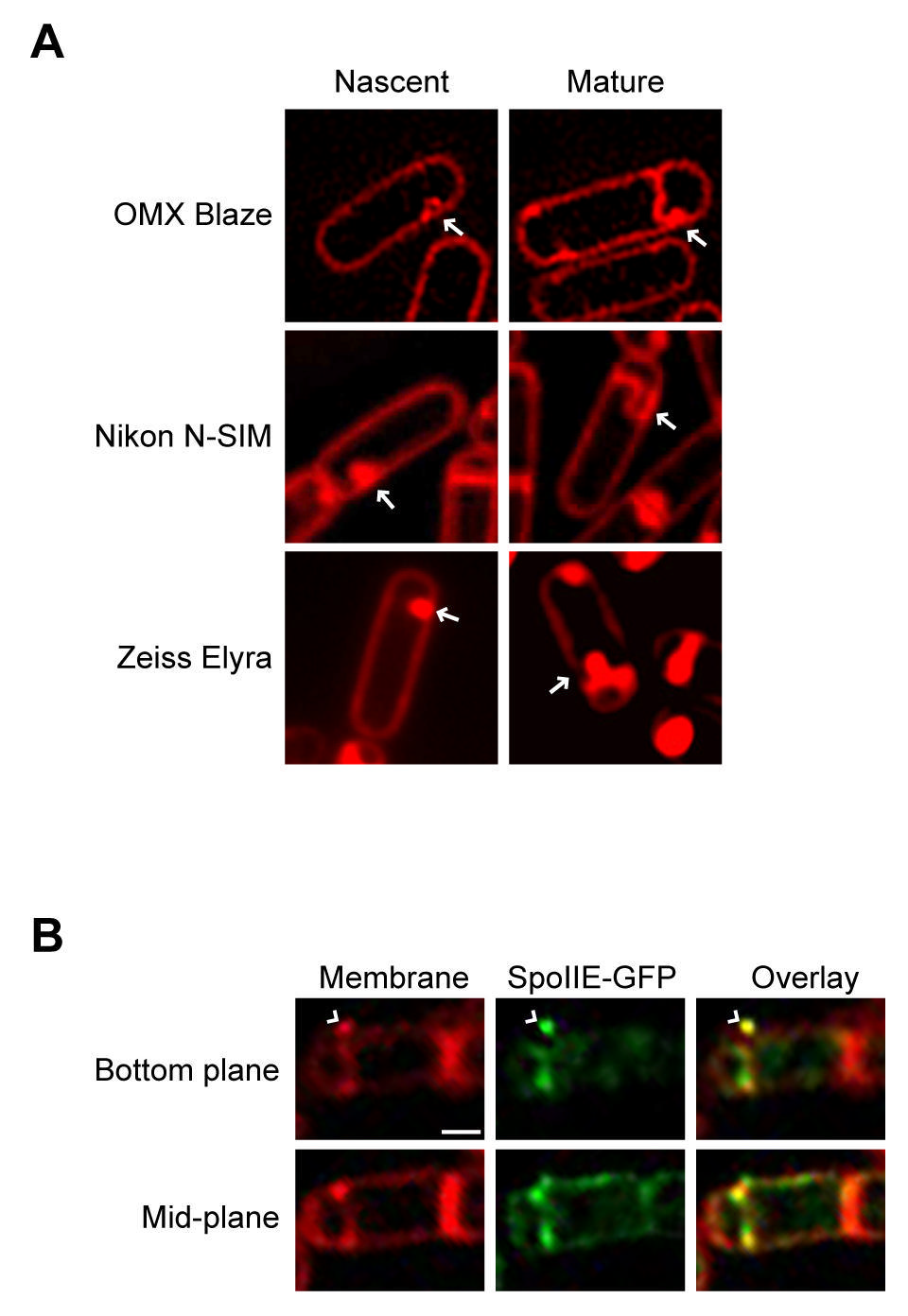

Supplement: Figure S7 — Super-resolution micrographs of sporulating B. subtilis cells. (A) Examples of types of deformation to the polar septum that were routinely observed using the lowest laser power available when viewing the cells using several commercial SIM setups: DeltaVision OMX Blaze (top row), Nikon N-SIM (middle row), or Zeiss Elyra (bottom row) at either nascent (left column) or mature (right column) polar septa. Arrows indicate the site of deformation. (B) Localization of SpoIIE-GFP in sporulating ΔspoIID ΔspoIIM cells (strain PE274) observed using MSIM. Internal calibration of fluorescence from red and green channels using a bead that fluoresces in both channels (arrowhead) as viewed at (top) a plane close to the coverslip or at (bottom) an intermediate plane. Scale bar: 0.5 µm. (TIF) [file pgen.1004526.s007.tif]

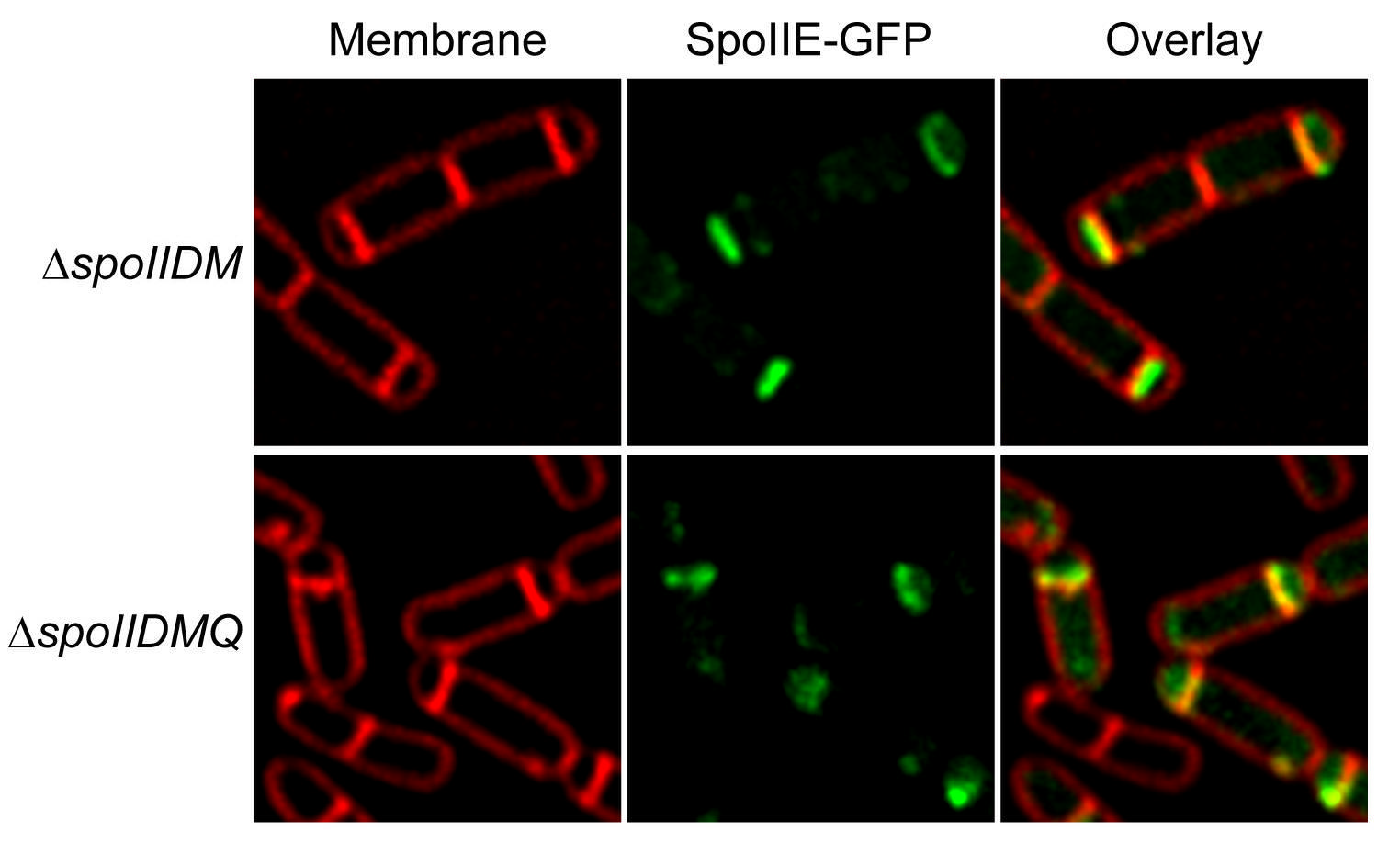

Supplement: Figure S8 — Localization of SpoIIE-GFP in the absence of SpoIIQ and engulfment. Subcellular localization of SpoIIE-GFP in mutant cells arrested at the flat septum stage before the onset of engulfment, 1.5 h after the induction of sporulation, in the presence (above, strain PE274) or absence (below, strain PE368) of SpoIIQ. (TIF) [file pgen.1004526.s008.tif]

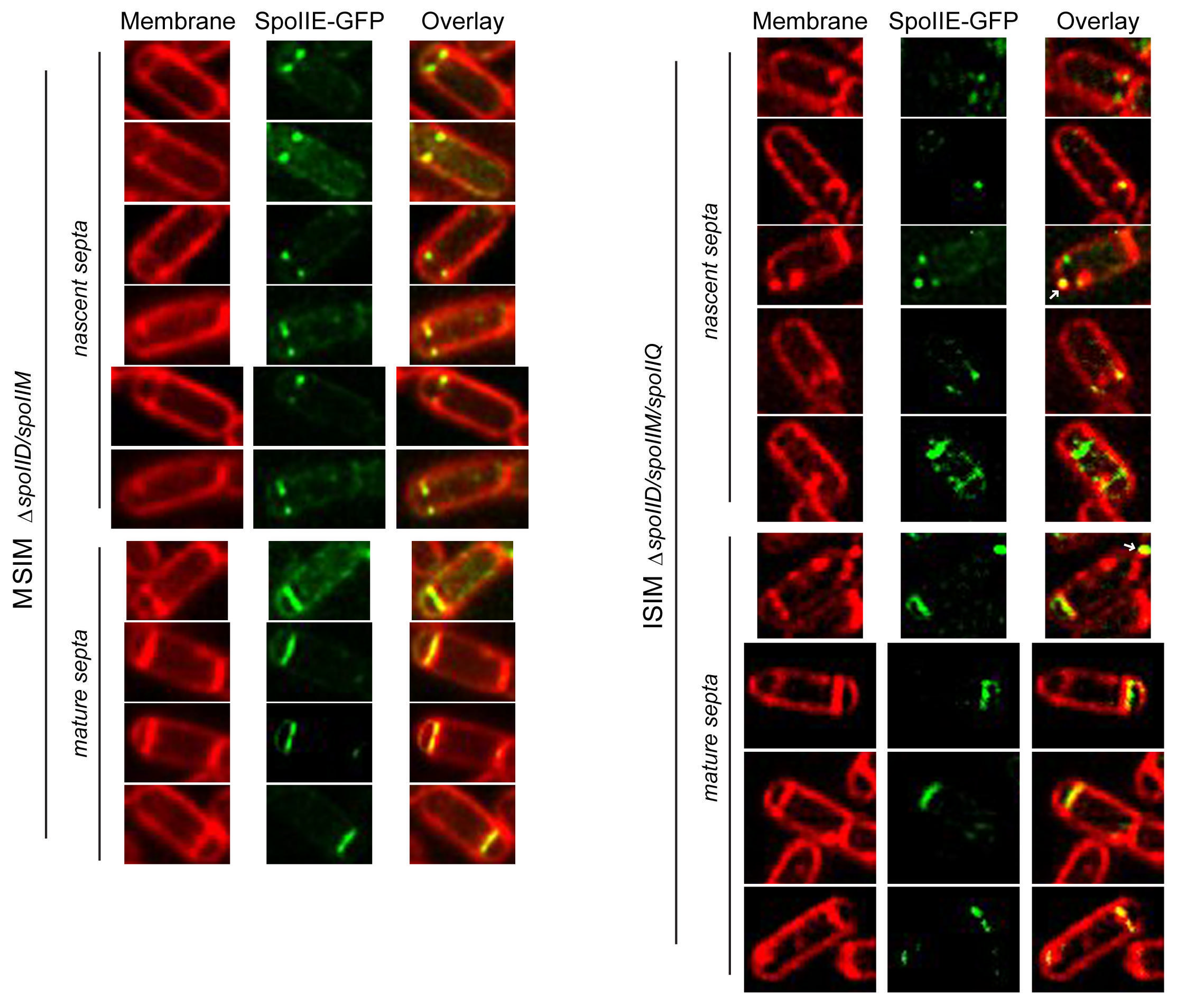

Supplement: Figure S9 — Gallery of sporulating cells displaying forespore-biased localization of SpoIIE-GFP. Subcellular localization of SpoIIE-GFP in mutant cells arrested at the flat septum stage before the onset of engulfment, 1.5 h after the induction of sporulation, in the presence (left, strain PE274) or absence (right, strain PE368) of SpoIIQ, visualized using either the MSIM or ISIM super-resolution technique. Arrows indicate fluorescent beads that were visible at mid-plane used for image registry. (TIF) [file pgen.1004526.s009.tif]

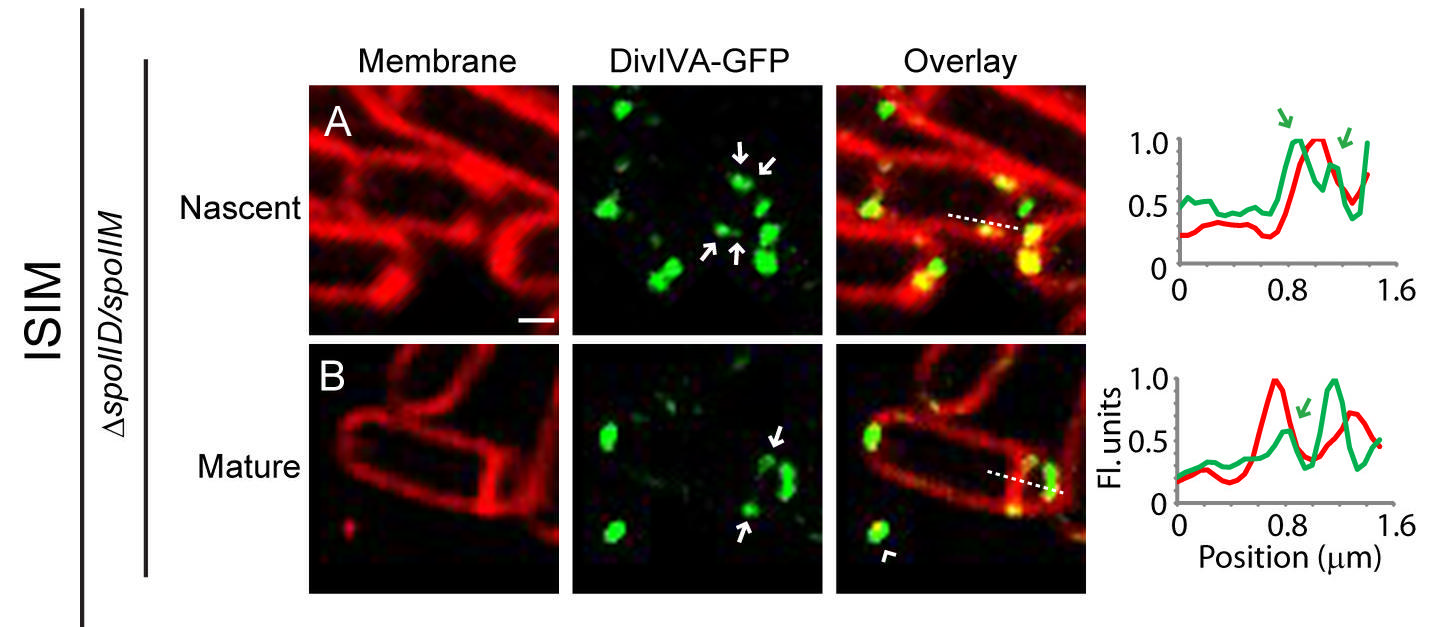

Supplement: Figure S10 — Preferential localization of DivIVA-GFP to the forespore side of the polar septum. Localization of DivIVA-GFP in sporulating ΔspoIID ΔspoIIM cells (strain PE275) displaying (A) nascent or (B) mature polar septa visualized using ISIM. Arrowhead indicates a fluorescent bead used for image registry that was visible at mid-plane. Linescan analyses of normalized fluorescence intensity along the axis of the dashed line in both channels at the selected polar septa are shown at the right; green arrows indicate the fluorescence from the GFP signal indicated with white arrows in the micrographs. Scale bar: 0.5 µm. (TIF) [file pgen.1004526.s010.tif]
