# Supplementary material for: Asymmetric Division and Differential Gene Expression during a Bacterial Developmental Program Requires DivIVA
Source: PLoS Genet. 2014 Aug 7;10(8):e1004526. doi: 10.1371/journal.pgen.1004526 (PMC4125091; doi:10.1371/journal.pgen.1004526)
Supplement: Table S1 — Bacillus subtilis strains used in this study. (DOC) [file pgen.1004526.s011.doc]

| **Table S1. *Bacillus subtilis* strains used in this study.** | | |
| --- | --- | --- |
| Strain | Genotype | Source |
| PY79 | Prototrophic derivative of *B. subtilis* 168 | [S1] |
| KR604 | *amyE::P_divIVA_-divIVA-linker-GFP cat* |  |
| PE177 | *amyE::P_divIVA_-divIVA-linker-CFP cat; thrC::P_spac_-yfp-ftsZ erm* | *thrC::P_spac_-yfp-ftsZ erm* [S2] |
| PE303 | *bkdB::Tn917::amyE::cat::P_spoIIG_-gfp spc::erm* | *bkdB::Tn917::amyE::cat* (Amy Camp); *amyE:: P_spoIIG_-gfp spc::erm* [S3] |
| PE304 | *bkdB::Tn917::amyE::cat::P_spoIIG_-gfp spc::erm; ∆divIVAΩdivIVA-FLAG-ssrA^E.coli^ cat* |  |
| PE329 | *bkdB::Tn917::amyE::cat::P_spoIIG_-gfp spc::erm; amyE::P_spank_-sspB^E.coli^ spc* | *amyE::P_spank_-sspB^E.coli^ spc* [52] |
| PE330 | *bkdB::Tn917::amyE::cat::P_spoIIG_-gfp spc::erm; ∆divIVAΩdivIVA-FLAG-ssrA^E.coli^ cat; amyE::P_spank_-sspB^E.coli^ spc* |  |
| PE305 | *amyE::P_spoIIG_-gfp spc::erm* |  |
| PE306 | *∆minCD::spc::cat;*  *amyE::P_spoIIG_-gfp spc::erm* | *∆minCD::spc* [S4] |
| PE307 | *∆divIVA::spc;*  *amyE::P_spoIIG_-gfp spc::erm* |  |
| PE318 | *∆minCD::spc::cat; ∆divIVA::spc*  *amyE::P_spoIIG_-gfp spc::erm* |  |
| PE339 | *∆racA::tet; amyE::P_spoIIG_-gfp spc::erm* | *∆racA::tet* (Dan Kearns) |
| PE340 | *∆minCD::spc::cat; ∆racA::tet*  *amyE::P_spoIIG_-gfp spc::erm* |  |
| MF3595 | *amyE::P2_ftsAZ_-lacZ cat* | Masaya Fujita [S5] |
| PE294 | *amyE::P2_ftsAZ_-lacZ cat; ∆minCD::spc* |  |
| PE295 | *amyE::P2_ftsAZ_-lacZ cat; ∆divIVA::erm* | *∆divIVA::erm* [40] |
| PE296 | *amyE::P2_ftsAZ_-lacZ cat; ∆minCD::spc; ∆divIVA::erm* |  |
| PE80 | *amyE::P_spoIIQ_-gfp spc* | From strain PE128 [S6] |
| PE81 | ∆*divIVA::erm; amyE::P_spoIIQ_-gfp spc* |  |
| PE259 | *∆minCD::spec::cat; amyE::P_spoIIQ_-gfp spc* |  |
| PE260 | *∆minCD::spec::cat; ∆divIVA::erm; amyE::P_spoIIQ_-gfp spc* |  |
| PE292 | *amyE::P_xyl_-gfp-zapA cat::tet; thrC::P_spoIIA_-mCherry erm* | *amyE::P_xyl_-gfp-zapA cat* [54]; *thrC::P_spoIIA_-mCherry erm* (Masaya Fujita) |
| PE320 | *amyE::P_xyl_-gfp-zapA cat::tet; ∆divIVA::spc;*  *thrC::P_spoIIA_-mCherry erm* |  |
| PE319 | *amyE::P_xyl_-gfp-zapA cat::tet; ∆minCD::spc::cat; thrC::P_spoIIA_-mCherry erm* |  |
| PE325 | *amyE::P_xyl_-gfp-zapA cat::tet; ∆divIVA::spc; ∆minCD::spc::cat; thrC::P_spoIIA_-mCherry erm* |  |
| PE118 | *spoIIE-gfp kan* | From strain SB201 [87] |
| KR610 | ∆*spoIIE::tet* |  |
| PE266 | *amyE:: P_divIVA_-divIVA-linker-GFP cat; ∆spoIIE::tet* |  |
| PE148 | *spoIIE-gfp kan; amyE:: P_divIVA_-divIVA-FLAG cat* |  |
| PE130 | *amyE::P_hyperspank_-spoIIE-GFP spc* |  |
| PE133 | *∆divIVA::erm; amyE::P_hyperspank_-spoIIE-GFP spc* |  |
| PE224 | *∆minCD::spc::cat; amyE::P_hyperspank_-spoIIE-GFP spc* |  |
| PE225 | *∆minCD::spc::cat; ∆divIVA::erm amyE::P_hyperspank_-spoIIE-GFP spc* |  |
| PE122 | *∆divIVA::erm; spoIIE-gfp kan* |  |
| PE138 | *∆minCD::spc; spoIIE-gfp kan* |  |
| PE141 | *∆minCD::spc; ∆divIVA::erm; spoIIE-gfp kan* |  |
| PE274 | *∆spoIID::cat::tet; ∆spoIIM::Tn917ΩHU287::erm; spoIIE-gfp kan* | From strain PE53 [S6] |
| PE275 | *∆spoIID::cat::tet; ∆spoIIM::Tn917ΩHU287::erm; amyE::divIVA-linker-GFP cat* |  |
| PE300 | *amyE::P_spoIIQ_-lacZ cat* | From strain AHB881 [S7] |
| PE321 | *amyE::P_spoIIQ_-lacZ cat; ∆minCD::spc* |  |
| PE322 | *amyE::P_spoIIQ_-lacZ cat; ∆divIVA::erm* |  |
| PE327 | *amyE::P_spoIIQ_-lacZ cat; ∆minCD::spc; ∆divIVA::erm* |  |
| PE301 | *amyE::P_spoIIE_-lacZ cat* | From strain NB606 (Neils Bradshaw) |
| PE323 | *amyE::P_spoIIE_-lacZ cat; ∆minCD::spc* |  |
| PE324 | *amyE::P_spoIIE_-lacZ cat; ∆divIVA::erm* |  |
| PE328 | *amyE::P_spoIIE_-lacZ cat; ∆minCD::spc; ∆divIVA::erm* |  |
| RL1275 | *∆sigF::erm* | [S8] |
| PE196 | *∆minCD::spc::cat; ∆sigF::erm* |  |
| PE199 | *∆divIVA::spc; ∆sigF::erm* |  |
| PE198 | *∆minCD::spc::cat; ∆sigF::erm; ∆divIVA::spc* |  |
| PE369 | *amyE::P_spac_-ftsZ-mCherry spc; spoIIE-gfp kan* | *amyE::P_spac_-ftsZ-mCherry spc* [S9] |
| PE375 | *amyE::P_hyperspank_-spoIIE-FLAG spc* |  |
| PE362 | *∆spo0A::kan* | From strain FC378, Losick lab |
| KR620 | *∆minCD::spc::cat* |  |
| KR543 | *∆divIVA::spc* | [S10] |
| PE308 | *∆minCD::spc::cat ∆divIVA::spc* |  |
| PE290 | *amyE::P_xyl_-gfp-zapA cat::tet* | From strain FG347 [9] |
| PE388 | *bkdB::Tn917::amyE::cat:: P_hyperspank_-spoVM-gfp spc; amyE:: P_divIVA_-divIVA-FLAG cat* | *amyE::P_hyperspank_-spoVM-gfp* [S10] |
| PE368 | *∆spoIID::cat::tet, ∆spoIIM::Tn917ΩHU287::erm; spoIIE-gfp kan; ∆spoIIQ::spc* | *∆spoIIQ::spc* [S11] |
| KR557 | *∆divIVA::spc; amyE::divIVA-FLAG cat* |  |
| KR600 | *∆divIVAΩdivIVA-FLAG-ssrA(E.coli) cat* |  |
| KR606 | *∆divIVA::erm amyE::divIVA-linker-GFP cat* |  |
| KR546 | *∆divIVA::erm* |  |
| PE180 | *∆spoIIE::tet; amyE::P_hyperspank_-spoIIE-GFP spc* |  |
| PE390 | *∆spoIIE::tet; amyE::P_hyperspank_-spoIIE-Flag spc* |  |
| KR541 | *amyE::P_hyperspank_-divIVA-gfp spc* | [42] |
|  |  |  |

**TABLE S1 REFERENCES**

S1. Youngman P, Perkins JB, Losick R (1984) Construction of a cloning site near one end of Tn917 into which foreign DNA may be inserted without affecting transposition in *Bacillus subtilis* or expression of the transposon-borne erm gene. *Plasmid* 12:1-9.

S2. Handler AA, Lim JE, Losick R (2008) Peptide inhibitor of cytokinesis during sporulation in *Bacillus subtilis*. *Mol Microbiol* 68:588-599.

S3. Fujita M, Losick R (2002) An investigation into the compartmentalization of the sporulation transcription factor sigmaE in *Bacillus subtilis*. *Mol Microbiol* 43:27-38.

S4. Levin PA, Shim JJ, Grossman AD (1998) Effect of minCD on FtsZ ring position and polar septation in *Bacillus subtilis*. *J Bacteriol* 180:6048-6051.

S5. Fukuchi K*, et al.* (2000) The essential two-component regulatory system encoded by yycF and yycG modulates expression of the *ftsAZ* operon in *Bacillus subtilis*. *Microbiology* 146 ( Pt 7):1573-1583.

S6. Eichenberger P, Fawcett P, Losick R (2001) A three-protein inhibitor of polar septation during sporulation in *Bacillus subtilis*. *Mol Microbiol* 42:1147-1162.

S7. Camp AH, Losick R (2009) A feeding tube model for activation of a cell-specific transcription factor during sporulation in *Bacillus subtilis*. *Genes Dev* 23:1014-1024.

S8. van Ooij C, Losick R (2003) Subcellular localization of a small sporulation protein in *Bacillus subtilis*. *J Bacteriol* 185:1391-1398.

S9. dos Santos VT, Bisson-Filho AW, Gueiros-Filho FJ (2012) DivIVA-mediated polar localization of ComN, a posttranscriptional regulator of *Bacillus subtilis*. *J Bacteriol* 194:3661-3669.

S10. Ramamurthi KS, Lecuyer S, Stone HA, Losick R (2009) Geometric cue for protein localization in a bacterium. *Science* 323:1354-1357.

S11. Londono-Vallejo JA, Frehel C, Stragier P (1997) SpoIIQ, a forespore-expressed gene required for engulfment in *Bacillus subtilis*. *Mol Microbiol* 24:29-39.
